# Making graphs compact by lossless contraction

**DOI:** 10.1007/s00778-022-00731-7

**Published:** 2022-02-19

**Authors:** Wenfei Fan, Yuanhao Li, Muyang Liu, Can Lu

**Affiliations:** 1 University of Edinburgh, Edinburgh, UK; 2 Shenzhen Institute of Computing Sciences, Shenzhen, China; 3 BDBC, Beihang University, Beijing, China

**Keywords:** Graph data management, Graph contraction, Graph algorithms, Incremental computation

## Abstract

This paper proposes a scheme to reduce big graphs to small graphs. It contracts obsolete parts and regular structures into supernodes. The supernodes carry a synopsis $S_\mathcal {Q}$ for each query class $\mathcal {Q}$ in use, to abstract key features of the contracted parts for answering queries of $\mathcal {Q}$. Moreover, for various types of graphs, we identify regular structures to contract. The contraction scheme provides a compact graph representation and prioritizes up-to-date data. Better still, it is generic and lossless. We show that the same contracted graph is able to support multiple query classes at the same time, no matter whether their queries are label based or not, local or non-local. Moreover, existing algorithms for these queries can be readily adapted to compute exact answers by using the synopses when possible and decontracting the supernodes only when necessary. As a proof of concept, we show how to adapt existing algorithms for subgraph isomorphism, triangle counting, shortest distance, connected component and clique decision to contracted graphs. We also provide a bounded incremental contraction algorithm in response to updates, such that its cost is determined by the size of areas affected by the updates alone, not by the entire graphs. We experimentally verify that on average, the contraction scheme reduces graphs by 71.9% and improves the evaluation of these queries by 1.69, 1.44, 1.47, 2.24 and 1.37 times, respectively.

## Introduction

There has been prevalent use of graphs in artificial intelligence, knowledge bases, search, recommendation, business transactions, fraud detection and social network analysis. Graphs in the real world are often big, *e.g.,* transaction graphs in e-commerce companies easily have billions of nodes and trillions of edges. Worse still, graph computations are often costly, *e.g.,* graph pattern matching via subgraph isomorphism is intractable (cf. [42]). These highlight the need for developing techniques for speeding up graph computations.

There has been a host of work on the subject, either by making graphs compact, *e.g.,* graph summarization [67] and compression [12, 82], or speeding up query answering by building indices [81]. The prior work often targets a specific class of queries, *e.g.,* query-preserving compression [37] and 2-hop labeling [25] are for reachability queries. In practice, however, multiple applications often run on the same graph at the same time. It is infeasible to switch compression schemes or summaries between different applications. It is also too costly to build indices for each and every query class in use.

Another challenge stems from obsolete data. As a real-life example, consider graphs converted from IT databases at a telecommunication company. The databases were developed in stages over years and have a large schema with hundreds of attributes. About 80% of the attributes were copied from earlier versions and have not been touched for years. No one can tell what these attributes are for, but no one has the gut to drop them in the fear of information loss. As a result, a large bulk of the graphs is obsolete. As another example, there are a large number of zombie accounts in Twitter. As reported by The New York Times, 71% of Lady Gaga’s followers are fake or inactive, and it is 58% for Justin Bieber. The obsolete data incur heavy time and space costs and often obscure query answers.

The challenges give rise to several questions. Is it possible to find a compact representation of graphs that is *generic* and *lossless*? That is, we want to reduce big graphs to a substantially smaller form. Moreover, using the *same* representation, we want to compute *exact answers* to *queries of different classes* *at the same time*. In addition, can the representation separate up-to-date data from obsolete components without loss of information? Can we adapt existing evaluation algorithms to the compact form, without the need for redeveloping the algorithms starting from scratch? Furthermore, can we efficiently and incrementally maintain the representation in response to updates to the original graphs?

**Contributions and organization**. In this paper, we propose a new approach to tackling these challenges, by extending the idea of graph contraction.

*(1) A contraction scheme* (Sect. 2). We propose a contraction scheme to reduce big graphs into smaller ones. It contracts obsolete components and regular structures into supernodes, and prioritizes up-to-date data. For each query class $\mathcal {Q}$, supernodes carry a synopsis $S_\mathcal {Q}$ that records key features needed for answering queries of $\mathcal {Q}$. As opposed to conventional graph summarization and compression, the scheme is generic and lossless. A contracted graph retains the same topological structure for all query classes $\mathcal {Q}$, and the same synopses $S_\mathcal {Q}$ work for all queries in the same class $\mathcal {Q}$. Only $S_\mathcal {Q}$ may vary for different query classes $\mathcal {Q}$. We identify regular structures to contract in different types of graphs, and develop a (parallel) contraction algorithm.

*(2) Proof of concept* (Sect. 3). We show that existing query evaluation algorithms can be readily adapted to contracted graphs. In a nutshell, we extend the algorithms to handle supernodes. When answering a query *Q* in $\mathcal {Q}$, we make use of the synopsis $S_\mathcal {Q}$ of a supernode if it carries sufficient information for answering *Q*, and decontract the supernode only when necessary. We pick five different query classes: subgraph isomorphism ($\mathsf {SubIso}$), triangle counting ($\mathsf {TriC}$), shortest distance ($\mathsf {Dist}$), connected component ($\mathsf {CC}$) and clique decision ($\mathsf {CD}$) based on the following dichotomies:

$$\circ $$
label-based queries ($\mathsf {SubIso}$) versus non-label based ones ($\mathsf {TriC}$, $\mathsf {Dist}$, $\mathsf {CC}$, $\mathsf {CD}$);
$$\circ $$
local queries ($\mathsf {SubIso}$, $\mathsf {TriC}$, $\mathsf {CD}$) versus non-local ones ($\mathsf {Dist}$, $\mathsf {CC}$); and
$$\circ $$
various degrees of topological constraints ($$\mathsf {Dist}$$ $$\prec $$ $$\mathsf {CC}$$ $$\prec $$ $$\mathsf {TriC}$$$$\prec $$ $$\mathsf {CD}$$$$\prec $$ $$\mathsf {SubIso}$$).

We show how easy to adapt existing algorithms for these query classes to contracted graphs, without increasing their complexity. Better still, all these queries can be answered *without decontraction of topological structures* except some supernodes for obsolete parts.

* (3) Incremental contraction* (Sect. 4). We develop an incremental algorithm for maintaining contracted graphs in response to updates to original graphs. Such updates may change both the topological structures and timestamps (obsolete data). We show that the algorithm is *bounded* [77], *i.e.,* it takes at most $O(|{\mathsf {AFF}} |^2)$ time, where $|{\mathsf {AFF}} |$ is the size of areas affected by updates, not the size of the entire (possibly big) graph. We parallelize the algorithm to scale with large graphs.

* (4) Empirical evaluation* (Sect. 5). Using 10 real-life graphs, we experimentally verify the following. On average, (a) the contraction scheme reduces graphs by 71.9%, up to $86.0\%$. (b) Contraction makes $\mathsf {SubIso}$, $\mathsf {TriC}$, $\mathsf {Dist}$, $\mathsf {CC}$ and $\mathsf {CD}$ 1.69, 1.44, 1.47, 2.24 and 1.37 times faster, respectively. (c) The total space cost of our contraction scheme for the five accounts only for 12.6% of indices for $\mathsf {TurboIso}$ [44], $\mathsf {HINDEX}$ [75], $\mathsf {PLL}$ [4] and $\mathsf {RMC}$ [68]. It is 9.0% when $\mathsf {kNN}$ [92] also runs on the same graph. The synopses for each take $9.7\%$ of the space. Hence, the scheme is scalable with the number of applications on the same graph. (d) Contracting obsolete data improves the efficiency of conventional queries and temporal queries by 1.64 and 1.78 times on average, respectively. (e) Our (incremental) contraction scheme scales well with graphs, *e.g.,* it takes 33.1s to contract graphs of 1.8B edges and nodes with 20 cores.

We survey related work in Sect. 6 and identify research topics for future work in Sect. 7.

## A graph contraction scheme

In this section, we first present the graph contraction scheme (Sect. 2.1). We then identify topological components to contract for different types of real-life graphs (Sect. 2.2). Moreover, we develop a contraction algorithm (Sect. 2.3) and its parallelization (Sect. 2.4).

**Fig. 1:**
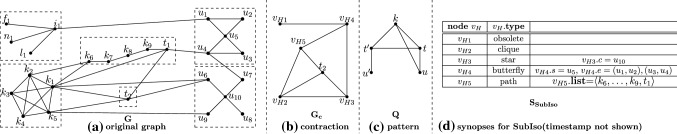
Graph contraction

**Preliminaries**. We start with basic notations.

* Graphs*. Assume two infinite sets $\Theta$ and $\Gamma$ for labels and timestamps, respectively. We consider undirected graphs $G = (V, E, L, T)$, where (a) *V* is a finite set of nodes, (b) $E\subseteq V \times V$ is a bag of edges, (c) for each node $v \in V$, *L*(*v*) is a label in $\Theta$; and (d) *T* is a partial function such that for each node $v \in V$, if *T*(*v*) is defined, it is a timestamp in $\Gamma$ that indicates the time when *v* or its adjacent edges were last updated.

* Queries*. A *graph query* is a computable function from a graph *G* to another object, *e.g.,* a Boolean value, a number, a graph, or a relation. For instance, a *graph pattern matching* query is a graph pattern *Q* to find the set of subgraphs in *G* that are isomorphic to pattern *Q*, denoted by *Q*(*G*). A *query class* $\mathcal {Q}$ is a set of queries of the same “type,” *e.g.,* all graph pattern queries. We also refer to $\mathcal {Q}$ as an *application*. In practice, multiple applications run on the same graph *G* *simultaneously*.

### Contraction scheme

A *graph contraction scheme* is a triple $\langle f_C, \mathcal {S}, f_D \rangle$, where (1) $f_C$ is a *contraction function* such that given a graph *G*, $G_c = f_C(G)$ is a graph deduced from *G* by contracting certain subgraphs *H* into supernodes $v_H$; we refer to *H* as the *subgraph contracted to* $v_H$, and $G_c$ as the *contracted graph* of *G* by $f_C$; (2) $\mathcal {S}$ is a set of *synopsis functions* such that for each query class $\mathcal {Q}$ in use, there exists $S_\mathcal {Q}\in \mathcal {S}$ that annotates each supernode $v_H$ of $G_c$ with a *synopsis* $S_\mathcal {Q}(v_H)$; and (3) $f_D$ is a *decontraction function* that restores each supernode $v_H$ in $G_c$ to its contracted subgraph *H*.

#### Example 1

Graph *G* in Fig. 1a is a fraction of Twitter network. A node denotes a user (*u*), a tweet (*t*), a keyword (*k*), or a feature of a user such as id (*i*), name (*n*), number of followers (*f*) and link to other accounts of the same user in other social networks (*l*). An edge indicates the following: (1) $(u,u')$, a user follows another; (2) (*u*, *t*), a user posts a tweet; (3) $(t,t')$, a tweet retweets another; (4) (*t*, *k*), a tweet tags a keyword; (5) $(k,k')$, two keywords are highly related; (6) (*u*, *k*), a user is interested in a keyword; (7) (*i*, *l*), a user has a feature; or (8) (*i*, *f*), a user has *f* followers.

In *G*, subgraphs in dashed rectangles are contracted into supernodes, yielding a contracted graph $G_c$ shown in Fig. 1b. Synopses $S_{\mathsf {SubIso}}$ for $\mathsf {SubIso}$ are shown in Fig. 1d and are elaborated in Sect. 3.1. $\square$

Before we formally define functions $f_C, f_D$ and synopsis $\mathcal {S}$, observe the following.

(1) The contraction scheme is *generic*. (a) Note that $f_C, G_c$ and $f_D$ are *application independent*, *i.e.,* they remain the same no matter what query classes $\mathcal {Q}$ run on the contracted graphs. (b) While $\mathcal {S}$ is application dependent, it is *query independent*, *i.e.,* all queries $Q \in \mathcal {Q}$ use the same synopses annotated by $S_\mathcal {Q}$.

(2) The contraction scheme is *lossless* due to synopses $\mathcal {S}$ and decontraction function $f_D$. As shown in Sect. 3, an existing algorithm $\mathcal {A}$ for a query class $\mathcal {Q}$ can be readily adapted to contracted graph and computes exact query answers.

We next give the details of $f_C, \mathcal {S}$ and $f_D$. We aim to strike a balance between space cost and query evaluation cost. When a graph is *over-contracted*, *i.e.,* when the subgraphs contracted to supernodes are too large or too small, the decontraction cost goes up although the contracted graph $G_c$ may take less space. Moreover, the more detailed synopses are, the less likely decontraction is needed, but the higher space overhead is incurred.

* (1) Contraction function*. Function $f_C$ contracts subgraphs in *G* into supernodes in $G_c$. To simplify the discussion, we contract the following basic structures.

(a) *Obsolete component*: a connected subgraph consisting of nodes with timestamps earlier than threshold $t_0$.

(b) *Topological component*: a subgraph with a regular structure, *e.g.,* clique, star, path and butterfly.

Different types of graphs have different regular substructures, *e.g.,* cliques are ubiquitous and effective in social networks while paths are only effective in road networks. In Sect. 2.2, we will identify what regular structures *H* to contract in different types of graphs.

We contract subgraphs with the number of nodes in the range $[k_l,k_u]$ to avoid over-contraction (see Sects. 2.3 and 5 for the choices).

Contraction function $f_C$ maps each node *v* in graph *G* to a supernode in contracted graph $G_c$, which is either a supernode $v_H$ if *v* falls in one of the subgraphs *H* in (a) or (b), or node *v* itself otherwise.

In Example 1, function $f_C$ maps nodes in each dashed rectangle to its corresponding supernode, *e.g.,* $f_C(i_1) = f_C(n_1) = f_C(f_1) = f_C(l_1) = v_{H1}$, $f_C(k_1) = \ldots = f_C(k_5) = v_{H2}$ and $f_C(t_2)=t_2$.

Obsolete components help us prioritize up-to-date data, and topological ones reduce unnecessary checking when answering queries. As shown in Sect. 5, on average the first three regular structures and obsolete components contribute 18.3%, 14.9%, 2.8% and 63.1% to the contraction ratio, and speeds up query answering by 1.61, 1.44, 1.04 and 1.71 times, respectively.

* (2) Contracted graph*. For a graph *G*, its *contracted graph by* $f_C$ is $G_c$ = $f_C(G)$ = $(V_c, E_c, f_C')$, where (a) $V_c$ is a set of supernodes mapped from *G* as remarked above; (b) $E_c \subseteq V_c \times V_c$ is a bag of superedges, where a *superedge* $(v_{H1}, v_{H2}) \in E_c$ if there exist nodes $v_1$ and $v_2$ such that $f_C(v_1)=v_{H1}$, $f_C(v_2)=v_{H2}$ and $(v_1, v_2) \in E$; and (c) $f_C'$ is the reverse function of $f_C$, *i.e.,* $f_C'(v_H)=\{(v,L(v))\ |\ f_C(v)=v_H\}$.

In Example 1, function $f_C'$ maps each supernode in contracted graph $G_c$ of Fig. 1b back to the nodes in the corresponding rectangle in Fig. 1a, *e.g.,* $f_C'(v_{H1})$ = $\{(i_1$, id), ($n_1$, name), ($f_1$, follower), ($l_1$, link)$\}$.

Intuitively, the reverse function $f_C'$ recovers the contracted nodes and their associated labels, while the decontraction function $f_D$ restores the topological structures of the contracted subgraphs.

* (3) Synopsis*. For each query class $\mathcal {Q}$ in use, a synopsis function $S_\mathcal {Q}$ is in $\mathcal {S}$, to retain features necessary for answering queries in $\mathcal {Q}$. For instance, when $\mathcal {Q}$ is the class of graph patterns, at each supernode $v_H$, $S_\mathcal {Q}(v_H)$ consists of the type of $v_H$ and the most distinguished features of $f_D(v_H)$, *e.g.,* the central node of a star and the sorted node list of a path. We will give more details about $S_\mathcal {Q}$ in Sect. 3. As will also be seen there, $f_C'$ and synopses $S_\mathcal {Q}$ taken together often suffice to answer queries in $\mathcal {Q}$, without decontraction.

Note that not every synopsis $S_\mathcal {Q}$ has to reside in memory. We load $S_\mathcal {Q}$ to memory only if its corresponding application $\mathcal {Q}$ is currently in use.

*(4) Decontraction*. Function $f_D$ restores contracted subgraphs. For *supernode* $v_H$, $f_D(v_H)$ restores the edges between the nodes in $f_C'(v_H)$, *i.e.,* the subgraph induced by $f_C'(v_H)$. For *superedge* $(v_{H1}, v_{H2})$, $f_D(v_{H1}, v_{H2})$ recovers the edges between $f_C'(v_{H1})$ and $f_C'(v_{H2})$.

That is, the contracted subgraphs and edges are not dropped. They can be restored by $f_D$ when necessary. In light of $f_D$, the scheme is guaranteed lossless.

For example, decontraction function $f_D$ restores the subgraph in Fig. 1a from supernodes, *e.g.,* $f_D(v_{H3})$ is a star with central node $u_{10}$ and leaves $u_6$, $u_7$, $u_8$ and $u_9$. It also restores edges from superedges, *e.g.,* $f_D(v_{H2}, v_{H5})=\{(t_1, k_1), (k_1, k_6), (k_2, k_6)\}$.

### Identifying regular structures

We now identify what regular structures to contract for *different types* of real-life graphs.

**Different types of graphs**. We investigated the following 10 different types of graphs: (1) social graphs: $\mathsf {Twitter}$ [70] and $\mathsf {LiveJournal}$ [94]; (2) communication networks: $\mathsf {WikiTalk}$ [62]; (3) citation networks: $\mathsf {HepTh}$ [63] and $\mathsf {Patent}$ [63]; (4) Web graphs: $\mathsf {Google}$ [64] and $\mathsf {NotreDame}$ [5]; (5) knowledge graphs: $\mathsf {DBpedia}$ [61] and $\mathsf {WordNet}$ [71]; (6) collaboration networks: $\mathsf {DBLP}$ [2] and $\mathsf {Hollywood}$ [15]; (7) biomedical graphs: $\mathsf {Mimic}$ [51]; (8) economic networks: $\mathsf {Poli}$ [80]; (9) chemical graphs: $\mathsf {Enzymes}$ [80]; and (10) road networks: $\mathsf {Traffic}$ [1].

**Regular structures**. For a certain type of graphs *G*, we apply a subgraph mining model ${{\mathcal {M}}}$ to *G*. It returns a set of frequent subgraphs ${{\mathcal {M}}}(G) = \{g_1, g_2, ...\}$ of *G* together with the *support* of each $g_i$. Support metrics may vary in different mining models, *e.g.,* $\mathsf {GRAMI}$ [33] adopts *minimum image based* metric [19]. We pick subgraphs whose supports are above a threshold $t_s$.

As an example, we adopt a subgraph miner $\mathsf {GRAMI}$ [33] as ${{\mathcal {M}}}$. $\mathsf {GRAMI}$ discovers all the frequent subgraphs in *G* that have a support above a predefined threshold, which are then manually inspected. We pick $g_i$’s with at least 4 nodes to avoid over-contraction.

**Fig. 2:**
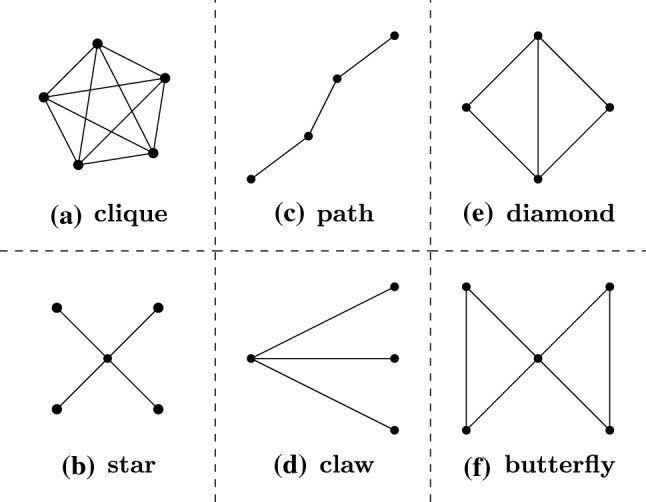
Frequent regular structures

As shown in Fig. 2, we found the following 6 structures in the 10 types of graphs: (a) clique: a fully-connected graph; (b) star: a single central node with neighbors; (c) path: a sequence of connected nodes with no edges between the head and tail (its two endpoints); (d) claw: a special star in which the central node has exactly 3 neighbors, denoted as its *leaves*; claws are quite frequent and are hence treated separately; (e) diamond: two triangles that share two endpoints; and (f) butterfly: two triangles sharing a single node.

Note that within these structures *H*, the only edges allowed are those that form *H*. Moreover, edges are allowed from each node in *H* to nodes outside of *H*. The only exception is that for a path, only the two endpoints can connect to other nodes in the graph.

**Table 1:** Common structures in different types of graphs

Graph type	Regular structure
Social graphs	Clique, star, diamond, butterfly, path
Communication networks	Star
Citation networks	Clique, star, diamond, butterfly
Web graphs	Star, clique, diamond
Knowledge graphs	Star, claw
Collaboration networks	Clique, star, diamond
Biomedical graphs	Star, clique, path
Economic networks	Star
Chemical graphs	Claw, path
Road networks	Star, claw, path

We summarize how these structures appear in the 10 *types* of graphs in Table 1, ordered by supports and importance from high to low. Note that different graphs have different frequent regular substructures. Cliques, stars and diamonds often occur in social graphs, while in road networks, stars, claws and paths are frequent.

Note that frequent pattern mining is conducted *once for each type* of graphs offline, *not* for each input graph. For instance, we always contract cliques, stars, diamonds, butterflies and paths for social graphs.

### Contraction algorithm

**Fig. 3:**
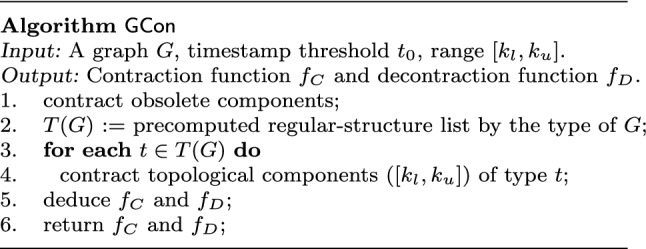
Algorithm $${\mathsf {GCon}} $$

We next present an algorithm to contract a given graph *G*, denoted as $\mathsf {GCon}$ and shown in Fig. 3.

A tricky issue is that the contracted graphs depend on the order on the regular structures contracted. For example, if we contract diamonds first in the Twitter graph $G_0$ of Fig. 1a, then it contracts $\{t_2, k_1, k_5, k_3\}$ as a diamond; after this there are no cliques in $G_0$. In contrast, if cliques are contracted first, then $\{k_1, k_2, k_3, k_4, k_5\}$ is extracted. As suggested by ${{\mathcal {M}}}$, cliques “dominate” in social graphs and hence should be “preserved” when contracting $G_0$.

We adopt a *deterministic* order to ensure that important structures are contracted earlier and hence preserved. We order the importance of different types of regular structures in a graph *G* by their supports: the higher the support is, the more important the topology is. We denote by *T*(*G*) its ordered set of regular structures to contract in Table 1. Note that *T*(*G*) is determined by *the type* of *G*, *e.g.,* social graphs, and is learned *once offline* regardless of individual *G*.

Given a graph *G*, algorithm $\mathsf {GCon}$ first contracts all obsolete data into components to prioritize up-to-date data. Each *obsolete component* is a connected subgraph that contains only nodes with timestamps earlier than a threshold $t_0$. It is extracted by bounded breadth-first-search ($\mathsf {BFS}$) that stops at non-obsolete nodes. The remaining nodes are then either contracted into topological components, or are left as singletons.

Putting these together, we present the main driver of algorithm $\mathsf {GCon}$ in Fig. 3. Given a graph *G*, a timestamp threshold $t_0$ and range $[k_l,k_u]$, it constructs functions $f_C$ and $f_D$ of the contraction scheme. It first contracts nodes with timestamps earlier than $t_0$ into obsolete components (line 1). It then recalls the list *T*(*G*) of topological components to contract based on the type of graph *G* (line 2). Next, $\mathsf {GCon}$ contracts topological components into supernodes following order *T*(*G*), and deduces $f_C$ and $f_D$ accordingly (lines 3-5). Each topological component consists of only uncontracted nodes. More specifically, it does the following.

(1) It extracts a clique by repeatedly selecting an un-contracted node that connects to all selected ones, subject to pre-selected size bounds $k_l$ and $k_u$ (see below).

(2) It extracts a star by first picking a central node $v_c$, and then repeatedly selecting an un-contracted node as a leaf that is (a) connected to $v_c$ and (b) disconnected from all selected leaves, again subject to $k_l$ and $k_u$.

(3) For paths, it first extracts intermediate nodes having only two neighbors that are not linked by an edge. It then finds a path consisting of only the intermediate nodes, along with two neighbors of the endpoints.

(4) For diamonds, it first selects an edge (*u*, *v*) and then picks *x* and *y* that are (a) connected to both *u* and *v*, and (b) pairwise disconnected.

(5) For butterflies, it first selects a node *v* that has a degree at least 4. It then checks whether there exist four neighbors *u*, *x*, *y*, *z* of node *v* such that exactly (*u*, *x*, *v*) and (*y*, *z*, *v*) form two triangles.

(6) For claws, it selects nodes with exactly 3 neighbors, and there is no edge between any two neighbors.

As remarked earlier, the remaining nodes that cannot be contracted into any component as above are treated as singleton, *i.e.,* mapped to themselves by $f_C$.

#### Example 2

Assume that timestamp threshold $$t_0$$ for graph *G* of Fig. 1a is larger than timestamps of nodes $$i_1$$, $$n_1$$, $$f_1$$ and $$l_1$$, but is smaller than those of remaining nodes. Algorithm $$\mathsf {GCon}$$ works as follows. (1) It first triggers bounded $$\mathsf {BFS}$$, and contracts $$i_1$$, $$n_1$$, $$f_1$$ and $$l_1$$ into an obsolete component $$v_{H1}$$ in $$G_c$$. (2) Since *G* is a social network, it contracts clique, star, diamond, butterfly and path in this order. (3) It builds a clique $$v_{H2}$$ with nodes $$k_1$$, ..., $$k_5$$. (4) It picks $$u_{10}$$ and $$u_5$$ as central nodes for a star, and makes a star $$v_{H3}$$ consisting of $$u_6,u_7,u_8,u_9,u_{10}$$. Nodes $$u_5, u_1, u_3$$ cannot make a star due to lower bound $$k_l=4$$. (5) No diamond exists. (6) It picks $$u_{5}$$ as central node for a butterfly and makes a butterfly $$v_{H4}$$. (7) It finds $$k_7$$, $$k_8$$ and $$k_9 $$ as candidate intermediate nodes for paths, and contracts them into a path $$v_{H5}$$ with endpoints $$k_6$$ and $$t_1$$. (8) Node $$t_2$$ is left as a singleton, and is mapped to itself by $$f_C$$. $$\square $$

*Range* $\underline{[}k_l,k_u]$. We contract an (obsolete/topological) component *H* such that the number of its nodes is in the range $[k_l, k_u]$. The reason is twofold. (1) If *H* is too small, a contracted graph would have an excessive number of supernodes; this leads to over-contraction with high overhead for possible decontraction and low contraction ratio. Thus, we set a lower bound $k_l$. (2) We set an upper bound $k_u$ to avoid overlarge components and excessive superedge decontraction. We experimentally find that the best $k_l$ and $k_u$ are 4 and 500, respectively.

Diamonds, butterflies and claws have a fixed size with 4, 5 and 4 nodes, respectively, in the range above.

* Complexity*. Algorithm $\mathsf {GCon}$ takes at most $O(|G|^2)$ time. Indeed, (1) obsolete components can be contracted in *O*(|*G*|) time via edge-disjoint bounded ${\mathsf {BFS}}$s; (2) paths can be built in *O*(|*G*|) time; (3) it takes *O*(|*G*|) time to contract each clique and $O(|G|^2)$ time for all cliques; and (4) similarly, the other regular structures can be contracted in $O(|G|^2)$ time.

**Properties**. Observe the following about the contraction scheme. (1) It is *lossless* and is able to compute exact query answers. (2) It is *generic* and supports multiple applications on the same contracted graph at the same time. This is often necessary. For instance, on average 10 classes of queries run on a graph simultaneously in GDB benchmarks [32]. (3) It *prioritizes up-to-date data* by separating it from obsolete data. (4) It improves performance. (a) As discussed in Sect. 5, $|G_c| \ll |G|$. In particular, each obsolete component is contracted into a single supernode. (b) Decontraction is often not needed. As shown in Sect. 3, none of $\mathsf {SubIso}$, $\mathsf {CD}$, $\mathsf {TriC}$, $\mathsf {Dist}$ and $\mathsf {CC}$ needs to decontract any topological component, and for $\mathsf {TriC}$, $\mathsf {Dist}$ and $\mathsf {CC}$, even obsolete components do not need decontraction.

### Parallel contraction algorithm

We next parallelize algorithm $\mathsf {GCon}$, denoted by $\mathsf {PCon}$, to speed up the contraction process. Note that contraction is conducted once offline, and is then incrementally maintained in response to updates (Sect. 4).

**Parallel setting**. Assume a master (processor) $M_0$ and *n* workers (processors) $P_1, \ldots , P_n$. Graph *G* is partitioned into *n* fragments $F_1, \ldots , F_n$ by an edge-cut partitioner [17, 55], and the fragments are distributed to *n* workers $P_1, \ldots , P_n$, respectively. We adopt the $\mathsf {BSP}$ model [88], which separates iterative computations into supersteps and synchronizes states after each superstep.

**Fig. 4:**
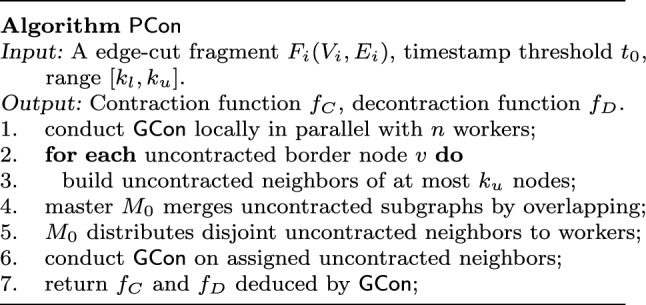
Algorithm $${\mathsf {PCon}} $$

**Parallel contraction algorithm** $\mathsf {PCon}$. As shown in Fig. 4, the idea of $\mathsf {PCon}$ is to leverage data-partitioned parallelism. $\mathsf {PCon}$ first conducts $\mathsf {GCon}$ locally on each fragment in parallel, and then contracts uncontracted “border nodes,” *i.e.,* nodes with edges crossing fragments, by building neighbors of at most $k_u$ uncontracted nodes, referred to as *uncontracted neighbors*, which are subgraphs with uncontracted nodes.

More specifically, algorithm $\mathsf {PCon}$ works as follows.

(1) All workers run $\mathsf {GCon}$ on its local fragment in parallel (line 1), since after all, each fragment $F_i$ is a graph itself.

In contrast with single-thread $\mathsf {GCon}$, workers do not contract mirror nodes, *i.e.,* nodes assigned to other fragments with edges linked to the local fragment. Adopting edge-cut partition, each node of *G* is assigned to a single fragment and is contracted at most once during $\mathsf {GCon}$.

(2) $\mathsf {PCon}$ contracts “border nodes” (line 2-3). For each border node *v*, if *v* is not contracted, $\mathsf {PCon}$ builds it uncontracted neighbors. Such neighbors are identified in parallel, coordinated by master $M_0$.

(3) Master $M_0$ merges overlapped neighbors into one, and distributes disjoint ones to *n* workers (line 4-5). In this way, $\mathsf {PCon}$ reduces communication cost and speeds up the process when contracting border nodes.

(4) Each worker contracts its assigned uncontracted-neighbors of border nodes, in parallel (line 6).

One can verify that each node *v* in *G* is contracted into at most one supernode $v_H$. The graph $G_c$ contracted by $\mathsf {PCon}$ may be slightly different from that of $\mathsf {GCon}$ since border nodes may be contracted in different orders. One can fix this by repeating steps (1)–(4) for each of topological components following the order *T*(*G*). Nonetheless, we experimentally find that the differences are not substantial enough to worth the extra cost. Moreover, the contracted graphs of $\mathsf {PCon}$ are ensured *compact*, *i.e.,* they cannot be contracted further.

## Proof of concept

In this section, we show that existing query evaluation algorithms can be readily adapted to the contracted graphs. As a proof of concept, we pick five query classes: (1) graph pattern matching $\mathsf {SubIso}$ via subgraph isomorphism (labeled queries with locality); (2) triangle counting $\mathsf {TriC}$ (un-labeled queries with locality); (3) shortest distance $\mathsf {Dist}$ (un-labeled and non-local queries); (4) connected component $\mathsf {CC}$ (un-labeled queries without locality); and (5) clique decision $\mathsf {CD}$ (un-labeled queries with locality). Among these, subgraph isomorphism and clique decision are intractable (cf. [42]).

Informally, when answering a query $Q \in \mathcal {Q}$, we check whether the synopsis $S_Q(v_H)$ at a supernode $v_H$ has enough information for *Q*; it uses $S_Q(v_H)$ directly if so; otherwise it decontracts superedges adjacent to $v_H$ or restores the subgraph of $v_H$ via decontraction function $f_D$. As will be seen shortly, $S_Q(v_H)$ often provides enough information to process *Q* at $v_H$ as a whole or safely skip $v_H$. Thus, it suffices to answer queries in the five classes by decontracting superedges, without decontracting any topological components. Here *decontraction* $f_D(v_{H1}, v_{H2})$ of a superedge $(v_{H1},v_{H2})$ restores the edges between $f_C'(v_{H1})$ and $f_C'(v_{H2})$ (Sect. 2).

The main result of this section is as follows.

### Theorem 1

Using **linear** synopsis functions,

(1) for each of $\mathsf {SubIso}$ and $\mathsf {CD}$, there are existing algorithms that can be adapted to compute exact answers on contracted graphs $G_c$, which decontract only supernodes of obsolete components and superedges between supernodes, **not any topological components**;

(2) for $\mathsf {TriC}$ and $\mathsf {Dist}$, there are existing algorithms that can be adapted to $G_c$ and decontract no supernodes, **neither topological nor obsolete components**; and

(3) for $\mathsf {CC}$, there are existing algorithms that can be adapted to $G_c$ and decontract **neither supernodes (topological and obsolete) nor superedges**. $\square$

Below we provide a constructive proof for Theorem 1 by adapting existing algorithms of the five query classes to contracted graphs one by one.

### Graph pattern matching with contraction

We start with graph pattern matching ($\mathsf {SubIso}$).

**Preliminaries**. We first review basic notations.

* Pattern*. A *graph pattern* is defined as a graph *Q* = ($V_Q$, $E_Q$, $L_Q$), where (1) $V_Q$ is a set of *pattern nodes*, (2) $E_Q$ is a set of *pattern edges*, and (3) $L_Q$ is a function that assigns a label $L_Q(u)$ to each $u\in V_Q$.

We also investigate *temporal patterns* (*Q*, *t*), where *Q* is a pattern as above and *t* is a given timestamp.

To simplify the discussion, we consider connected patterns *Q*. This said, our algorithm can be adapted to disconnected ones. We denote by *u*, *v* pattern nodes in pattern *Q*, and by *x*, *y* nodes in graph *G*. A *neighbor* of node *v* is a node such that $(u,v) \in E_Q$.

* Pattern matching*. A *match* of pattern *Q* in graph *G* is a subgraph $G' = (V', E', L', T')$ of *G* that is isomorphic to *Q*, *i.e.,* there exists a *bijective function* $h: V_Q \rightarrow V'$ such that (1) for each node $u \in V_Q$, $L_Q(u) = L(h(u))$; and (2) $e = (u, u')$ is an edge in pattern *Q* iff (if and only if) $(h(u), h(u'))$ is an edge in graph *G*. We denote by *Q*(*G*) the set of all matches of pattern *Q* in graph *G*.

A *match* of a temporal pattern (*Q*, *t*) in graph *G* is a match $G'$ in *Q*(*G*) such that for each node *v* in $G'$, $T'(v) > t$, *i.e.,* a match of (conventional) pattern *Q* in which all nodes have timestamps later than *t*. We denote by *Q*(*G*, *t*) all matches of (*Q*, *t*) in *G*.

The *graph pattern matching problem*, denoted by $\mathsf {SubIso}$, is to compute, given a pattern *Q* and a graph *G*, the set *Q*(*G*) of matches. Similarly, the *temporal matching problem* is to compute *Q*(*G*, *t*) for a given temporal pattern (*Q*, *t*) and a graph *G*, denoted by ${\mathsf {SubIso}} _t$.

Graph pattern matching is widely used in graph queries [6, 40, 79, 90] and graph dependencies [36, 39].

Note that (1) patterns *Q* are *labeled*, *i.e.,* nodes are matched by labels. Moreover, (2) *Q* has the *locality*, *i.e.,* for any match $G'$ of *Q* in *G* and any nodes $v_1$, $v_2$ in $G'$, $v_1$ and $v_2$ are within $d_Q$ hops when treating $G'$ as undirected. Here $d_Q$ is the *diameter* of *Q*, *i.e.,* the maximum shortest distance between any two nodes in *Q*.

The decision problem of pattern matching is $\mathsf {NP}$-complete (cf. [42]); similarly for temporal matching. A variety of algorithms have been developed for $\mathsf {SubIso}$, notably $\mathsf {TurboIso}$ [44] with indices and $\mathsf {VF2}$ [28] without index. Both $\mathsf {TurboIso}$ and $\mathsf {VF2}$ can be adapted to contracted graphs as characterized in Theorem 1.

We give a constructive proof for $\mathsf {TurboIso}$, because (1) it is one of the most efficient algorithms for subgraph isomorphism and is followed by other $\mathsf {SubIso}$ algorithms *e.g.,* [14, 78], and (2) it employs indexing to reduce redundant matches; by adapting $\mathsf {TurboIso}$ we show that the indices for $\mathsf {SubIso}$ can be inherited by contracted graphs, *i.e.,* contraction and indexing complement each other. The same algorithm works for temporal matching. The proof for $\mathsf {VF2}$ is simpler (not shown).

Below we first present synopses for $\mathsf {SubIso}$ (Sect. 3.1.1), which are the same for both $\mathsf {VF2}$ and $\mathsf {TurboIso}$. We then show how to adapt algorithm $\mathsf {TurboIso}$ to contracted graphs (Sect. 3.1.2)

#### Contraction for $\mathsf {SubIso}$

Observe that topological components have regular structures. The idea of synopses is to store the types and key features of regular structures so that we could check pattern matching without decontracting any supernodes of topological components.

The synopsis of a supernode $v_H$ for query class $\mathsf {SubIso}$ is defined as follows:

$$\circ $$
clique: $$v_H.{\mathsf {type}} $$ = clique;
$$\circ $$
star: $v_H.{\mathsf {type}}$ = star, $v_H.c$ records its central node;
$$\circ $$
path: $v_H.{\mathsf {type}}$ = path, $v_H.{\mathsf {list}} = \langle u_1, \ldots , u_{|v_c|}\rangle$, storing all the nodes on the path in order;
$$\circ $$
diamond: $v_H.{\mathsf {type}}$ = diamond, $v_H.s_1$ and $v_H.s_2$ store the two share nodes of the two triangles;
$$\circ $$
butterfly: $v_H.{\mathsf {type}}$ =butterfly, $v_H.s$ records the node shared by the two triangles, and $v_H.e$ stores the two disjoint edges;
$$\circ $$
claw: $v_H.{\mathsf {type}}$ =claw, $v_H.c$ stores the central node and $v_H.s_i$ ($i \in [1, 3]$) record its three neighbors;
$$\circ $$
obsolete component: $v_H.{\mathsf {type}}$ = obsolete; and
$$\circ $$
each component maintains $v_H.t$ = ${\mathsf {max}} \{T(v)\ |\ v \in f'_C(v_H)\}$, *i.e.,* the largest timestamp of its nodes.

Node labels are stored in the reverse function $f_c'$ of the contraction function $f_c$ (see Sect. 2.1).

For instance, the synopsis $S_{\mathsf {SubIso}} (v_H)$ for each supernode $v_H$ in the contracted graph $G_c$ of Fig. 1b is given in Fig. 1d. Note that $S_{\mathsf {SubIso}}$ only stores the synopses of the regular structures contracted in a graph.

* Properties*. The synopses in $S_{\mathsf {SubIso}}$ have two properties. (1) Taken with the reverse function $f_C'$ of $f_C$, the synopsis of a supernode $v_H$ suffices to recover topological component *H* contracted to $v_H$. For instance, given the central node and leaf nodes, a star can be uniquely determined. As a result, no supernode decontraction is needed for topological components. (2) The synopses can be constructed during the traversal of *G* for constructing contracted graph $G_c$, as a byproduct.

**Fig. 5:**
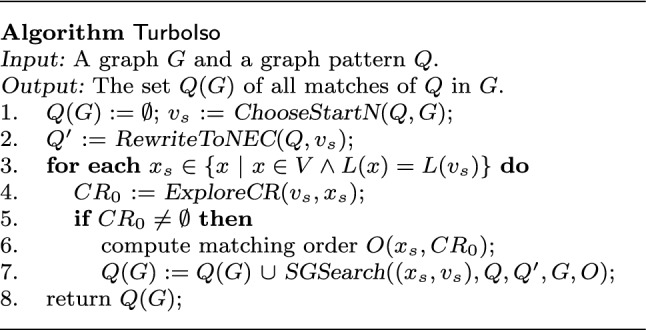
Algorithm $${\mathsf {TurboIso}} $$

We remark that the design of synopses needs domain knowledge. This said, (1) users only need to develop synopses for their applications in use, not exhaustively for all possible query classes; and (2) synopsis design is no harder than developing indexing structures.

#### Subgraph isomorphism

Below we first review algorithm $\mathsf {TurboIso}$ [44] and then show how to adapt $\mathsf {TurboIso}$ to contracted graphs.

$\underline{ {\mathsf {TurboIso}}}$. As shown in Fig. 5, given a graph *G* and a pattern *Q*, $\mathsf {TurboIso}$ computes *Q*(*G*) as follows. It first rewrites pattern graph *Q* into a tree $Q'$ by performing $\mathsf {BFS}$ from a start vertex $v_s$ (lines 1-2). Here each vertex in $Q'$ is a *neighborhood equivalence class* (NEC) that contains pattern nodes in *Q* having identically matching data vertices. Then, for each start vertex $x_s$ of each region, $\mathsf {TurboIso}$ constructs a candidate region ($CR_0$), *i.e.,* an index that maintains candidates for each NEC vertex in $Q'$, via $\mathsf {DFS}$ from $x_s$ (lines 3-4). If valid candidates are found, *i.e.,* $CR_0 \ne \emptyset$, $\mathsf {TurboIso}$ enumerates all possible matches that map $x_s$ to $v_s$ following a matching order *O* (lines 5-6). The matching order *O* is decided by sorting the leaf NEC vertices based on the number of their candidate vertices. It expands *Q*(*G*) with valid matches identified in the process (line 7).

**Fig. 6:**
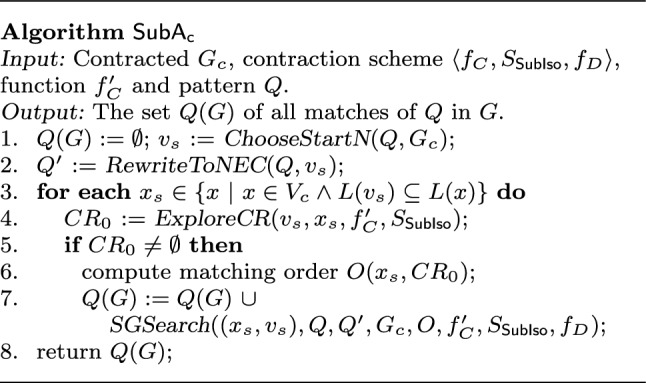
Algorithm $${\mathsf {SubA_{c}}} $$

**Algorithm** $\mathsf {SubA_{c}}$. $\mathsf {TurboIso}$ can be easily adapted to contracted graph $G_c$, denoted by $\mathsf {SubA_{c}}$. As shown in Fig. 6, $\mathsf {SubA_{c}}$ adopts the same logic as $\mathsf {TurboIso}$ except minor adaptations in $\mathsf {ExploreCR}$ (line 4) and $\mathsf {SGSearch}$ (line 7) to deal with supernodes. To see these, let *H* be the subgraph contracted to a supernode $v_H$.

(1) $\mathsf {ExploreCR}$. It adds a supernode $v_H$ as a candidate for a node *u* in *Q* if some node in $v_H$ can match *u*, which is checked by $S_{{\mathsf {SubIso}}}(v_H)$ and $f_C'(v_H)$. It also prunes $CR_0$ based on $v_H.{\mathsf {type}}$, *e.g.,* a node *u* in *Q* cannot match intermediate nodes on paths if *u* is in some triangle in *Q*; and *u* matches intermediate nodes on a path only if its degree is no larger than 2. No supernodes or superedges are decontracted.

(2) $\mathsf {SGSearch}$. Checking the existence of an edge (*x*, *y*) that matches edge $(v_x, v_y) \in Q$ is easy with synopses $S_{\mathsf {SubIso}}$ and functions $f_C'$ and $f_D$. Here *x* (resp. *y*) denotes a node in supernode $v_H=f_C(x)$ (resp. $v_H=f_C(y)$) in the candidates of $v_x$ (resp. $v_y$). When $f_C(x)=f_C(y)=v_H$, (a) if $v_H.{\mathsf {type}}$=star or claw, (*x*, *y*) exists only if $x=v_H.c$ or $y=v_H.c$; (b) if $v_H.{\mathsf {type}}$ = clique, (*x*, *y*) always exists; (c) if $v_H.{\mathsf {type}}$=path, (*x*, *y*) exists if *x* and *y* are next to each other in $v_H.{\mathsf {list}}$; (d) if $v_H.{\mathsf {type}}$=diamond, (*x*, *y*) exists if at least one of *x* and *y* is the shared node $v_H.s_1$ or $v_H.s_2$; and (e) if $v_H.{\mathsf {type}}$=butterfly, (*x*, *y*) exists if *x* and *y* are not endpoints of the two disjoint edges in $v_H.e$ simultaneously. Hence, no topological component is decontracted by $f_D$. (f) If $v_H.{\mathsf {type}}$=obsolete, it checks whether none of the labels in *Q* is in $f_C'(v_H)$; it safely skips $v_H$ if so, and decontracts $v_H$ by $f_D$ to check the existence of (*x*, *y*) otherwise. If *x* and *y* match distinct supernodes, it suffices to decontract superedge $(f_C(x), f_C(y))$ by $f_D$.

##### Example 3

Query *Q* in Fig. 1c is to find potential friendship between users based on the retweet and shared keywords in their posted tweets. Nodes *u* and $$u'$$ both have the same label *u*. Given *Q*, $$\mathsf {SubA_{c}}$$ first chooses *k* as the start node, to which only $$v_{H2}$$ and $$v_{H5}$$ can match. For $$v_{H2}$$, $$\mathsf {ExploreCR}$$ adds $$v_{H5}$$ and $$t_2$$ as candidates for *t* and $$t'$$, $$ v_{H3}$$ as candidate for *u*, and $$v_{H3}$$ and $$v_{H4}$$ as candidates for $$u'$$. Note that for obsolete supernode $$v_{H1}$$, none of the labels in *Q* is covered by $$f_C'(v_{H1})$$; hence, $$v_{H1}$$ can be safely skipped. $$\mathsf {SGSearch}$$ finds that $$t_2$$ matches *t* since there exists no edge between $$v_{H3}$$ and $$v_{H5}$$. Thus, it matches $$k,t,u,t',u'$$ with $$k_1, t_2,u_6,t_1,u_4$$.

Similarly for $v_{H5}$, $\mathsf {ExploreCR}$ adds $v_{H5}$ and $t_2$ as candidates for *t* and $t'$, $v_{H4}$ as candidate for *u*, and $v_{H3}$ and $v_{H4}$ for *u* and $u'$. Next, $\mathsf {SGSearch}$ finds that $u_4$ and $t_1$ match *u* and *t* by decontracting superedge $(v_{H3},v_{H4})$; then, $k_9$ matches *k*. However, since $k_9$ is an intermediate node of path $v_{H3}$, no match for $t'$ can be found. Hence, $k,t,u,t',u'$ match $k_1, t_2,u_6,t_1,u_4$. $\square$

* Analyses*. One can easily verify that $\mathsf {SubA_{c}}$ is correct since it has the same logic as $\mathsf {TurboIso}$ except that it incorporates pruning strategies. While they have the same worst-case complexity, $\mathsf {SubA_{c}}$ operates on $G_c$, much smaller than *G* (see Sect. 5); moreover, its $\mathsf {ExplorCR}$ saves traversal cost and $\mathsf {SGSearch}$ saves validation cost by pruning invalid matches.

**Temporal pattern matching**. Algorithm $\mathsf {SubA_{c}}$ can also take a temporal pattern (*Q*, *t*) as part of its input, instead of *Q*. The only major difference is at $CR_0$ construction (line 4), where a supernode $v_H$ is safely pruned if $v_H.t \le t$, when $v_H.{\mathsf {type}}$ is obsolete or not. It skips a match if it contains a node *v* with $T(v) \le t$.

### Triangle counting with contraction

We next study triangle counting [26, 47], which has been used in clustering [91], cycle detection [48] and transitivity [74]. In graph *G*, a *triangle* is a clique of three vertices. The *triangle counting problem* is to find the total number of triangles in *G*, denoted by $\mathsf {TriC}$.

Similar to $\mathsf {SubIso}$, $\mathsf {TriC}$ is local with diameter 1. In contrast, it consists of a single query and is not labeled.

We adapt algorithm $\mathsf {TriA}$ of [26] for $\mathsf {TriC}$ to contracted graphs, since it is one of the most efficient $\mathsf {TriC}$ algorithms [47], and it does not use indexing (as a different example from $\mathsf {TurboIso}$). We show that for $\mathsf {TriC}$, the adapted algorithm needs to decontract no supernodes, neither topological components nor obsolete parts.

#### Contraction for $\mathsf {TriC}$

Observe that contraction function $f_C$ on *G* is equivalent to node partition of *G*, such that two nodes are in the same partition if they are contracted into the same supernode. The idea of synopses for $\mathsf {TriC}$ is to pre-count triangles with at least two nodes in the same partition, without enumerating them. As will be seen shortly, this allows us to avoid supernode decontraction for both topological and obsolete components.

Consider a triangle (*u*, *v*, *w*) in *G* that is mapped to $G_c$ via $f_C$. We have the following cases.

(1) If $f_C(u)=f_C(v)=f_C(w)=v_H$, where supernode $v_H$ contracts a subgraph *H* with node set *V*(*H*), *i.e.,* when the three nodes of a triangle are contracted into the same supernode, then (a) when *H* is a clique, there are $$\left( {\begin{array}{c}|V(H)|\\ 3\end{array}}\right) $$ triangles inside *H*; (b) when *H* is a diamond or a butterfly, there are 2 triangles inside *H*; (c) when *H* is an obsolete component, then the number of triangles inside *H* can be pre-calculated, denoted by $t_H$; and (d) there are no triangles inside *H* otherwise.

(2) If $f_C(u)=f_C(v)=v_I$, $f_C(w) = v_J$, where $v_I$ and $v_J$ contract subgraphs *I* and *J*, respectively, *i.e.,* if two nodes of a triangle are contracted into the same supernode, then (a) when *I* is a clique, then *w* leads to $$\left( {\begin{array}{c}k\\ 2\end{array}}\right) $$ triangles, where *k* is the number of the neighbors of *w* in *I*. Denote by $t_w^I$ the number of such triangles in a clique neighbor *I* of *w*. (b) Subgraph *I* cannot be a path since intermediate nodes on a path are not allowed to connect to nodes outside *I*. (c) Otherwise, nodes *u* and *v* yield *k* triangles, where *k* is the number of common neighbors of *u* and *v* in *J*. We denote by $t_{u,v}^J$ the number of such triangles in a common neighbor *J* of *u* and *v*.

(3) If $f_C(u)=v_I$, $f_C(v)=v_J$, $f_C(w) = v_K$, *i.e.,* when the three nodes of a triangle are contracted into different supernodes, we count such triangles online and it suffices to decontract only superedges, not supernodes.

Synopsis $S_{\mathsf {TriC}} (v_H)$ of supernode $v_H$ for $\mathsf {TriC}$ extends $S_{\mathsf {SubIso}} (v_H)$ with an extra tag ${\mathsf {tc}}$, which records the number of triangles pre-calculated as above. More specifically, $v_H.{\mathsf {tc}}$ is computed as follows. Below we use *u* and *v* to range over nodes in *V*(*H*), *I* to range over clique neighbors of *u*, and *J* to range over common neighbors of *u*, *v*. We define $t_u^I$, $t_{H}$ and $t_{u,v}^J$ as above.

In a clique *H*, there are (1) $$\left( {\begin{array}{c}|V(H)|\\ 3\end{array}}\right) $$ triangles; (2) each node $u \in H$ has $t_u^I$ triangles with its clique neighbor *I*; hence, $$v_H.{\mathsf {tc}} =\left( {\begin{array}{c}|V(H)|\\ 3\end{array}}\right) +\Sigma _u\Sigma _I t_u^I$$. We can calculate $v_H.{\mathsf {tc}}$ similarly for other regular structures. Thus,

$$\circ $$
clique: $$v_H.{\mathsf {tc}} =\left( {\begin{array}{c}|V(H)|\\ 3\end{array}}\right) +\Sigma _u\Sigma _I t_u^I$$;
$$\circ $$
star: $$v_H.{\mathsf {tc}} =\Sigma _u\Sigma _I t_u^I+\Sigma _u\Sigma _J t_{v_H.c, u}^J$$;
$$\circ $$
path: $v_H.{\mathsf {tc}} =\Sigma _I t_{u_1}^I + \Sigma _I t_{u_{|V(H)|}}^I$, where $u_1$ and $u_{|V(H)|}$ are the first and last node on the path;
$$\circ $$
claw: $$v_H.{\mathsf {tc}} =\Sigma _u\Sigma _I t_u^I + \Sigma _{u,v}\Sigma _J t_{u,v}^J$$;
$$\circ $$
diamond and butterfly: $v_H.{\mathsf {tc}} =2+\Sigma _u\Sigma _I t_u^I + \Sigma _{u,v}\Sigma _J t_{u,v}^J$,
$$\circ $$
obsolete: $$v_H.{\mathsf {tc}} =t_H+\Sigma _u\Sigma _I t_u^I + \Sigma _{u,v}\Sigma _J t_{u,v}^J$$.

Synopses $S_{\mathsf {TriC}}$ also share the properties of $S_{\mathsf {SubIso}}$.

##### Example 4

In the contracted graph $G_c$ of Fig. 1b, only $v_{H2}$ contracts a clique, denoted by *I*. Synopsis $S_{\mathsf {TriC}} (v_H)$ of a supernode $v_H$ extends $S_{\mathsf {SubIso}} (v_H)$ with $v_H.{\mathsf {tc}}$: (1) for $v_{H1}$, (a) *H*1 contracted to $v_{H1}$ contains no triangles; thus, $t_{H1}=0$; (b) *I* is not a neighbor of any node *u* in *V*(*H*1); thus, $t_u^{I}=0$; and (c) nodes in *V*(*H*1) have no common neighbors, *i.e.,* no *J* exists for any connected $u,v \in V(H1)$; thus, $t_{u,v}^J=0$. Hence, $v_{H1}.{\mathsf {tc}} =0$. (2) For $v_{H2}$, $v_{H2}.{\mathsf {type}}$=clique, $|V(H2)|=5$ and no other supernodes in $G_c$ are cliques. Hence, $v_{H2}.{\mathsf {tc}} = 10$. (3) For $v_{H3}$, $u_6$ and $u_9$ have only 1 neighbor in clique *I*; thus, $t_u^I=0$; similarly, no *J* exists for any leaf *u* and $v_{H3}.c$; thus, $t_{v_{H3}.c, u}^J=0$. Hence, $v_{H3}.{\mathsf {tc}} =0$. (4) Similarly, $v_{H4}.{\mathsf {tc}} =2$, $v_{H5}.{\mathsf {tc}} =1$ and $t_2.{\mathsf {tc}} =1$. $\square$

#### Triangle counting

We now adapt algorithm $\mathsf {TriA}$ [26] to contracted graphs. The adapted algorithm is referred to as $\mathsf {TriA_{c}}$.

*Algorithm* $\underline{{\mathsf {TriA}}}$. Given a graph *G*, $\mathsf {TriA}$ assigns distinct numbers to all the nodes in *G*. It then enumerates triangles for each edge (*u*, *v*) by counting the common neighbors *w* of *u* and *v* such that $w < u$ and $w<v$.

**Algorithm** $\mathsf {TriA_{c}}$. On a contracted graph $G_c$ with superedges decontracted, $\mathsf {TriA_{c}}$ works in the same way as $\mathsf {TriA}$ except that at a supernode $v_H$ (for both topological and obsolete components), it simply accumulates $v_H.{\mathsf {tc}}$ without decontraction or enumeration. It only restores superedges when necessary.

##### Example 5

From synopsis $S_{\mathsf {TriC}}$, $\mathsf {TriA_{c}}$ directly finds 14 triangles. In $G_c$, it finds two additional triangles $(u_6, t_2, k_1)$ and $(t_1, t_2, k_1)$ by restoring superedges. Thus, it finds 16 triangles in *G*. No supernodes of either topological or obsolete components are decontracted. $\square$

* Analyses*. One can verify that $\mathsf {TriA_{c}}$ is correct since it counts all triangles in *G* once and only once. It speeds up $\mathsf {TriA}$ since it works on a smaller contracted $G_c$.

**Temporal triangle counting**. Algorithm $\mathsf {TriA_{c}}$ can be adapted to count triangles with timestamp later than a given time *t*. It prunes a supernode $v_H$ if $v_H.t \le t$, and drops a triangle if it has a node *v* with $T(v) \le t$.

### Shortest distance with contraction

We next study the shortest distance problem.

*Shortest distance*. Consider an undirected weighted graph $G = (V,E,L,T, W)$ with additional weight *W*; for each edge *e*, *W*(*e*) is a positive number for the length of the edge. In a graph *G*, a *path* *p* from $v_0$ to $v_k$ is a sequence $\langle v_0, v_1, \ldots , v_k \rangle$ of nodes such that $(v_i, v_{i+1}) \in E$ for all $0 \le i <k$. The length of a path $p=(v_0, \ldots , v_k)$ in *G* is simply ${\mathsf {sum}} _{i \in [1,k]}W(v_{i-1}, v_i)$.

The *shortest distance problem*, denoted by $\mathsf {Dist}$, is to compute, given a pair (*u*, *v*) of nodes in *G*, the shortest distance between *u* and *v*, denoted by *d*(*u*, *v*) [4, 25, 31].

Shortest distance has a wide range of applications, *e.g.,* socially-sensitive search [89, 93], influential community detection [9, 56] and centrality analysis [16, 18].

As opposed to $\mathsf {SubIso}$, shortest distance queries are *unlabeled*, *i.e.,* the value of a query answer *d*(*u*, *v*) does not depend on labels. In contrast with $\mathsf {SubIso}$ and $\mathsf {TriC}$, $\mathsf {Dist}$ is non-local, *i.e.,* there exists no *d* independent of the input graph *G* such that $d(u,v)<d$.

We adapt Dijkstra’s algorithm [31] to contracted graphs, denoted by $\mathsf {Dijkstra}$, which is one of the best known algorithms for $\mathsf {Dist}$. Just like $\mathsf {TriC}$, the adapted algorithm for $\mathsf {Dist}$ decontracts no supernodes, neither topological components nor obsolete parts.

#### Contraction for $\mathsf {Dist}$

A path between nodes *u* and *v* can be decomposed into (1) edges between supernodes, and (2) edges within a supernode. The idea of synopses for $\mathsf {Dist}$ is to pre-compute the shortest distances within supernodes to avoid supernode decontraction, for both topological and obsolete components. Edges between supernodes are recovered by superedge decontraction when necessary.

Suppose that $v_1$ and $v_2$ are nodes mapped to supernode $v_H$ by $f_C$, *i.e.,* $f_C(v_1)=f_C(v_2)=v_H$. We compute the shortest distance for $(v_1, v_2)$ within the subgraph *H* contracted to $v_H$, denoted by $d_{v_H}(v_1, v_2)$. The synopsis $S_{\mathsf {Dist}} (v_H)$ extends $S_{\mathsf {SubIso}} (v_H)$ with a tag ${\mathsf {dis}}$ that is a set of triples $(v_1, v_2, d_{v_H}(v_1, v_2))$ for a path between $v_1$ and $v_2$ within $v_{H}$, based on $v_H$.$\mathsf {type}$:

$$\circ $$
clique: $v_H.{\mathsf {dis}} = \{(v_1, v_2, d_{v_H}(v_1, v_2))\}$ for all pairs of $v_1, v_2{\in }f_C'(v_H)$;
$$\circ $$
path: $v_H.{\mathsf {dis}} =\{(u_1, u_{|f_C'(v_H)|},\Sigma _{1\le i < |f_C'(v_H)|}W(u_i, u_{i+1}))\}$, *i.e.,* it records the path itself;
$$\circ $$
diamond, butterfly and obsolete components: $v_H.{\mathsf {dis}} = \{(v_1, v_2, d_{v_H}(v_1, v_2))\ |\ v_1, v_2{\in }f_C'(v_H)\}$.

In practice, the number of nodes in most contracted subgraphs is far below the upper bound $k_u$. Indeed, diamonds and butterflies have a constant size, and we find that a clique (resp. star, path and obsolete component) typically contains 6.5 (resp. 7.3, 4.1 and 49.2) nodes. Hence, the size of a synopsis is fairly small. Note that the upper bound $k_u$ should be larger than typical sizes of components, since large components exist and may be more powerful for accelerating computations.

##### Example 6

Assume $W(u,v)=1$ for all edges (*u*, *v*) in graph *G* of Fig. 1a. Then, for supernodes in the contracted graph of Fig. 1b, (1) $v_{H1}.{\mathsf {dis}} =\{(i_1, f_1, 1), (i_1, n_1,1), (i_1, l_1, 1),(f_1, n_1, 2), (f_1, l_1,2), (n_1, l_1, 2)\}$; (2) $$v_{H2}.{\mathsf {dis}} =\{(k_i, k_j, 1) \ |\ $$ $1\le i<j\le 5\}$; (3) $v_{H4}.{\mathsf {dis}} = \{(u_1,u_2,1), (u_1,u_5,1)$, $(u_1,u_3,2)$, $(u_1,u_4,2), \ldots \}$; and finally, (4) $v_{H5}.{\mathsf {dis}} =\{(k_6, t_1, 4)\}$. $\square$

#### Shortest distance

We adapt algorithm $\mathsf {Dijkstra}$ [31] to contracted graphs $G_c$, and refer to the adapted algorithm as $\mathsf {DisA_{c}}$.

*Algorithm* $\underline{{\mathsf {Dijkstra}}}$. Given a graph *G* and a pair (*u*, *v*) of nodes, $\mathsf {Dijkstra}$ finds the shortest distances from *u* to nodes in *G* in ascending order, and terminates as soon as *d*(*u*, *v*) is determined. It maintains a set *S* of nodes whose shortest distances from *u* are known; it initializes distance estimates ${{\overline{d}}}(u)=0$, and ${\overline{d}}(w)=\infty$ for other nodes. At each step, $\mathsf {Dijkstra}$ moves a node *w* from $V \setminus S$ to *S* that has minimal ${{\overline{d}}}(w)$, and updates distance estimates of nodes adjacent to *w* accordingly.

**Algorithm** $\mathsf {DisA_{c}}$. $\mathsf {DisA_{c}}$ is the same as $\mathsf {Dijkstra}$ except minor changes to updating distance estimates. When moving a node *w* from $V \setminus S$ to *S*, suppose that $v_H$ is the supernode to which *w* is mapped, *i.e.,* $f_C(w)=v_H$. $\mathsf {DisA_{c}}$ updates distance estimates ${{\overline{d}}}(w')$ for $w' \in f_C'(v_H)$ as follows: (1) if $v_H.{\mathsf {type}}$ is clique, butterfly, diamond or obsolete, update ${{\overline{d}}}(w')$ by ${{\overline{d}}}(w)+d_{v_H}(w,w')$ using $v_H.{\mathsf {dis}}$; (2) if $v_H.{\mathsf {type}}$ = star or claw, update ${\overline{d}}(w')$ by ${{\overline{d}}}(w)+d_{v_H}(w,w')$, where $d_{v_H}(w,w')$ can be easily computed by synopsis; (3) if $v_H.{\mathsf {type}}$ = path, update ${{\overline{d}}}(w')$ by ${{\overline{d}}}(w)+d_{v_H}(w,w')$ for the other endpoint $w'$ using $v_H.{\mathsf {dis}}$; in these cases, no supernode (for topological or obsolete components) is decontracted. $\mathsf {DisA_{c}}$ updates ${{\overline{d}}}(w')$ by ${\overline{d}}(w)+W(w,w')$ for all edges $(w,w')$ where $f_C(w) \ne f_C(w')$, by decontracting superedge $(f_C(w), f_C(w'))$ at worst, in the same way as $\mathsf {Dijkstra}$.

##### Example 7

Given $\mathsf {Dist}$ query $(u_2, k_5)$ on the contracted graph $G_c$ of Fig. 1b, $\mathsf {DisA_{c}}$ works in the following steps: (1) initially, $S=\emptyset$, ${{\overline{d}}}(u_2)=0$, and ${{\overline{d}}}(v)=\infty$ for all other nodes; (2) $S=\{u_2\}$, ${{\overline{d}}}(u_1)={{\overline{d}}}(u_5) =1$, ${{\overline{d}}}(u_3) = {{\overline{d}}}(u_4) = 2$ by using $S_{\mathsf {Dist}} (v_{H4})$; (3) $S = \{u_2, u_1,u_5,u_3,u_4\}$, ${{\overline{d}}}(t_1) =3$ by edge $(u_4,t_1)$, and ${{\overline{d}}}(k_6) = {{\overline{d}}}(t_1) + d_{v_{H3}}(k_6,t_1)=7$ by $v_{H5}.{\mathsf {dis}}$; ${{\overline{d}}}(i_1) =2$ by edge $(u_1,i_1)$, and ${{\overline{d}}}(f_1)={\overline{d}}(n_1)={{\overline{d}}}(l_1)=3$ by $v_{H1}.{\mathsf {dis}}$; similarly, ${{\overline{d}}}(u_7)=3$ and ${{\overline{d}}}(u_{10})$ = 4, ${\overline{d}}(u_6)={{\overline{d}}}(u_8)={{\overline{d}}}(u_9)=5$ by making use of reverse function $f_C'$ and synopsis $S_{\mathsf {Dist}} (v_{H3})$ (note that $v_{H3}$ contracts a star); (4) $S=\{u_2, u_1, u_5, u_3, u_4, i_1, t_1, u_7\}$, ${{\overline{d}}}(t_2) = 4$ by edge $(t_1,t_2)$; and (5) $S = \{u_2, u_1, u_5, u_3, u_4, i_1, t_1, u_7, f_1, n_1, l_1, t_2\}$, ${{\overline{d}}}(k_1) = {{\overline{d}}}(k_3) = {{\overline{d}}}(k_5)=5$ by edges $(t_2,k_1)$, $(t_2,k_3)$, $(t_2,k_5)$. When $\mathsf {DisA_{c}}$ moves node $k_5$ to *S*, it gets $d(k_5)=5$. The algorithm returns $d(u_2, k_5)=5$. $\square$

* Analyses*. By induction on the length of shortest paths, one can verify that $\mathsf {DisA_{c}}$ is correct. In particular, for each node $w'$ in *G*, when ${{\overline{d}}}(w')$ is updated by a node *w* that is mapped to the same supernode, the update is equivalent to a series of $\mathsf {Dijkstra}$ updates. Moreover, $\mathsf {DisA_{c}}$ works on smaller contracted graphs $G_c$ and saves traversal cost inside contracted components without any decontraction, neither topological nor obsolete.

**Temporal shortest distance**. Similar to temporal $\mathsf {SubIso}$ and $\mathsf {TriC}$, we study *temporal* $\mathsf {Dist}$ *queries* (*u*, *v*, *t*), where (*u*, *v*) is a pair of nodes as in $\mathsf {Dist}$, and *t* is a timestamp. It is to compute the shortest length of paths *p* from *u* to *v* such that for each node *w* on *p*, $T(w) > t$.

Algorithm $\mathsf {DisA_{c}}$ can be easily adapted to temporal $\mathsf {Dist}$, by skipping nodes *v* with $T(v) \le t$. In particular, it safely ignores a supernode $v_H$ if $v_H.t \le t$.

### Connected component with contraction

We next study the connected component problem [29, 85]. In a graph *G*, a *connected component* is a maximal subgraph of *G* in which any two nodes are connected to each other via a path. The *connected component problem*, denoted as $\mathsf {CC}$, is to compute the set of pairs (*s*, *n*) for a given graph *G*, where (*s*, *n*) indicates that there are *n* connected components in *G* that consist of *s* nodes.

Given a graph *G*, $\mathsf {CC}$ returns the numbers of connected components of various sizes in *G*. Similar to $\mathsf {Dist}$, $\mathsf {CC}$ is a *non-local* query, *i.e.,* it has to traverse the entire graph when answering the query. It is also *un-labeled*, *i.e.,* labels have no impact on its query answer.

This form of $\mathsf {CC}$ is used in pattern recognition [45, 53], graph partition [86] and random walk [49].

We adapt algorithm $\mathsf {CCA}$ of [85] for $\mathsf {CC}$ to contracted graphs, since it is one of the most efficient $\mathsf {CC}$ algorithms. Better still, we show that the adapted algorithm decontracts neither supernodes nor superedges.

#### Contraction for $\mathsf {CC}$

The synopsis $S_{\mathsf {SubIso}}$ for $\mathsf {SubIso}$ suffices for us to answer $\mathsf {CC}$ queries. Observe that each subgraph *H* contracted to a supernode $v_H$ is connected, no matter whether *H* is a topological component or an obsolete component. We can regard a supernode $v_H$ as a whole when evaluating $\mathsf {CC}$ queries, and leverage $S_{\mathsf {SubIso}} (v_H)$ and $f_C'$ to compute the size of connected components. We need neither additional synopses nor any decontraction.

#### Connected component

We now adapt algorithm $\mathsf {CCA}$ [85] to contracted graphs. The adapted algorithm is referred to as $\mathsf {CCA_{c}}$.

*Algorithm* $\underline{{\mathsf {CCA}}}$. We first review how $\mathsf {CCA}$ works. (1) Starting from each unvisited node *v* in graph *G*, $\mathsf {CCA}$ performs a depth-first-search ($\mathsf {DFS}$) and collects all unvisited nodes reached in the traversal. These nodes are connected to *v* and are marked as visited. When no more nodes are unvisited, all visited nodes and *v* form a connected component. $\mathsf {CCA}$ records its size *s*. (2) After all nodes in *G* are visited, $\mathsf {CCA}$ groups connected components by size *s* and returns the aggregate (*s*, *n*).

**Algorithm** $\mathsf {CCA_{c}}$. On the contracted graph $G_c$, $\mathsf {CCA_{c}}$ works in the same way as $\mathsf {CCA}$ except that (1) it only performs $\mathsf {DFS}$ on $G_c$, without decontracting any supernodes or superedges; and (2) the size of each connected component is aggregated as the sum of the size $|f_C'(v_H)|$ of all supernodes $v_H$ in the component.

##### Example 8

On the contracted graph in Fig. 1b, $\mathsf {CCA_{c}}$ finds a connected component that consists of supernodes $v_{H1}, v_{H2}, v_{H3}, v_{H4}, v_{H5}$ and $t_2$. The size *s* of this component is simply the sum $|f_C'(v_{H1})|+\dots +|f_C'(v_{H5})|+|f_C'(t_2)|$, *i.e.,* $s = 25$. Since all the supernodes in $G_c$ have been visited, $\mathsf {CCA_{c}}$ outputs (25, 1). $\square$

* Analyses*. $\mathsf {CCA_{c}}$ is correct since it follows the same logic as $\mathsf {CCA}$ and all contracted subgraphs are guaranteed to be connected. The algorithm takes at most $O(|G_c|)$ time while $\mathsf {CCA}$ takes *O*(|*G*|) time. Since $G_c$ is much smaller than *G*, $\mathsf {CCA_{c}}$ always outperforms $\mathsf {CCA}$.

**Temporal connected component**. $\mathsf {CCA_{c}}$ can be adapted to compute connected components with timestamp later than a given time *t*, by skipping nodes *v* with $T(v) \le t$. It safely ignores a supernode $v_H$ if $v_H.t \le t$.

### Clique decision with contraction

We next study a decision problem for clique. A *clique* in a graph *G* is a subgraph *C* in which there are edges between any two nodes; it is a *k*-clique if the number of nodes in *C* is *k* (*i.e.,* $|V(C)|=k$) . We consider the *clique decision problem* [20, 57], denoted by $\mathsf {CD}$, to find whether there exists a *k*-clique in *G* for a given number *k*. $\mathsf {CD}$ is being widely used in community search [76], team formation [59] and anomaly detection [11, 65].

Similar to $\mathsf {Dist}$ and $\mathsf {CC}$, $\mathsf {CD}$ is un-labeled. In contrast with $\mathsf {Dist}$ and $\mathsf {CC}$, but similar to $\mathsf {SubIso}$, it is local, *i.e.,* all nodes in a clique are within 1 hop of each other.

The clique decision problem is known $\mathsf {NP}$-complete (cf. [42]). A variety of algorithms have been developed for $\mathsf {CD}$, notable $\mathsf {CDA}$ of [57], which we will adapt next.

#### Contraction for $\mathsf {CD}$

Observe the following. (1) Cliques in *G* contracted into supernodes in $G_c$ can help us find an initial maximum clique (see below). (2) The degree of a node can be used as an upper bound of the maximum clique containing it.

In light of these, we extend synopsis $S_{\mathsf {SubIso}} (v_H)$ with tags $\mathsf {cs}$ and $\mathsf {md}$. For a subgraph *H* that is contracted to a supernode $v_H$, the two tags record the maximum clique found in *H* and the maximum degree of the nodes in *H*, respectively. Specifically, $v_H.{\mathsf {cs}}$ is based on $v_H.{\mathsf {type}}$:

$$\circ $$
clique: $$v_H.{\mathsf {cs}} = |f_C'(v_H)|$$;
$$\circ $$
diamond and butterfly: $v_H.{\mathsf {cs}} = 3$;
$$\circ $$
star, path and claw: $v_H.{\mathsf {cs}} = 2$; and
$$\circ $$
obsolete component: we find a *k*-clique in an obsolete component online.

and $v_H.{\mathsf {md}}$ is by aggregation:

$$\circ $$
node *v*: $$v.{\mathsf {md}} = |\{u\ |\ (u,v)\in E\}|$$; and
$$\circ $$
supernode $$v_H$$: $$v_H.{\mathsf {md}} = \max \{v.{\mathsf {md}}\ |\ f_C(v)=v_H\}$$.

Synopses $S_{\mathsf {CD}}$ also share the properties of $S_{\mathsf {SubIso}}$.

##### Example 9

In the contracted graph $G_c$ of Fig. 1b, $S_{\mathsf {CD}} (v_H)$ extends $S_{\mathsf {SubIso}} (v_H)$ with tags $\mathsf {cs}$ and $\mathsf {md}$ as follows. Since $v_{H2}$ contracts a clique, $v_{H2}.{\mathsf {cs}} = 5$; $v_{H4}.{\mathsf {cs}} =3$ since $v_{H4}$ contracts a butterfly, and $v_H.{\mathsf {cs}} =2$ for supernodes $v_{H3}$ (star) and $v_{H5}$ (path). For tag $\mathsf {md}$, $v_{H1}.{\mathsf {md}} =i_1.{\mathsf {md}} =4$; similarly, $v_{H2}.{\mathsf {md}} =8$, $v_{H3}.{\mathsf {md}} =4$, $v_{H4}.{\mathsf {md}} =4$, $v_{H5}.{\mathsf {md}} =4$, and $t_2.{\mathsf {md}} =4$. $\square$

#### Clique decision

We adapt $\mathsf {CDA}$ [57] to $G_c$, denoted as $\mathsf {CDA_{c}}$.

* Algorithm* $\underline{{\mathsf {CDA}}}$. We first review $\mathsf {CDA}$. Given a graph *G*, algorithm $\mathsf {CDA}$ checks the existence of a *k*-clique in *G* by branch-and-bound. It branches from each node in *G*. Denote by *C* the current clique in the search, and by *P* the set of common neighbors of the nodes in *C*. $\mathsf {CDA}$ (1) bounds the search from *C* if $|C|+|P| <k$, or (2) branches from each node *u* in *P* to expand *C*. More specifically, it iteratively adds a node *u* from *P* to *C* and removes all those nodes in *P* that are not neighbors of *u*, enlarging *C* and shrinking *P* until *P* is empty. If $|C|\ge k$, then *C* contains a *k*-clique and $\mathsf {CDA}$ terminates with $\mathsf {true}$; it returns $\mathsf {false}$ if no *k*-clique is found after all branches are searched.

**Algorithm** $\mathsf {CDA_{c}}$. $\mathsf {CDA_{c}}$ adopts the same logic as $\mathsf {CDA}$ except the following: (1) it picks the maximum synopsis $v_H.{\mathsf {cs}}$ among all supernodes $v_H$ in $G_c$; a *k*-clique is found directly if $v_H.{\mathsf {cs}} \ge k$; and (2) it skips a supernode $v_H$ in $G_c$ if $v_H.{\mathsf {md}} < k-1$. Superedges adjacent to $v_H$ are skipped as well since no *k*-clique contains any node contracted to $v_H$. Otherwise, it checks the synopsis of $v_H$ if $v_H$ contracts a topological component, or restores obsolete component *H* contracted to $v_H$, to check cliques in the original graph *G*. Note that $\mathsf {CDA_{c}}$ initiates the search with the largest clique contracted, by checking the synopses. Hence, cliques play a more important role than the other regular structures for $\mathsf {CD}$.

##### Example 10

For query with $k=5$, by $S_{\mathsf {CD}} (v_{H2})$ of Fig. 1b, $\mathsf {CDA_{c}}$ finds a 5-clique and returns true.

For query with $k=6$, all supernodes except $v_{H2}$ are skipped by synopses. Their adjacent superedges are skipped as well. Since $v_{H2}$ only contracts a 5-clique, $\mathsf {CDA_{c}}$ fails to find a 6-clique and returns false. $\square$

*Analyses*. One can verify that $\mathsf {CDA_{c}}$ is correct since it follows the same logic as $\mathsf {CDA}$ except that it adopts pruning strategies that are possible because of the use of synopses. While the two algorithms have the same worst-case complexity, $\mathsf {CDA_{c}}$ starts with a supernode with a maximum clique and may find a *k*-clique directly; moreover, it skips a supernode as a whole by synopses, which reduces unnecessary search and validation.

**Temporal ***k*-**clique**. Algorithm $\mathsf {CDA_{c}}$ can be adapted to find a *k*-clique with timestamp later than a given time *t*, by skipping nodes *v* with $T(v) \le t$. Like $\mathsf {SubA_{c}}$ and $\mathsf {TriA_{c}}$, it safely ignores a supernode $v_H$ if $v_H.t \le t$.

## Incremental contraction

We next develop an incremental algorithm to maintain contracted graphs in response to updates $\Delta G$ to graphs *G*. We start with batch update $\Delta G$, which is a sequence of edge insertions and deletions. We formulate the problem (Sect. 4.1), present the incremental algorithm (Sects. 4.2–4.3), discuss vertex updates (Sect. 4.4), and parallelize the algorithm (Sect. 4.5).

### Incremental contraction problem

*Updates* to a graph *G*, denoted by $\Delta G$, consists of (1) node updates, *i.e.,* node insertions and deletions; and (2) edge updates, *i.e.,* edge insertions and deletions.

Given a contraction scheme $\langle f_C, \mathcal {S}, f_D\rangle$, a contracted graph $G_c=f_C(G)$, and updates $\Delta G$, the *incremental contraction problem*, denoted as $\mathsf {ICP}$, is to compute (a) changes $\Delta G_c$ to $G_c$ such that $G_c \oplus \Delta G_c = f_C(G \oplus \Delta G)$, *i.e.,* to get the contracted graph of the updated graph $G \oplus \Delta G$, where $G_c \oplus \Delta G_c$ applies $\Delta G_c$ to $G_c$; (b) the updated synopses of affected supernodes; and (c) functions $f_C \oplus \Delta f_C$ and $f_D \oplus \Delta f_D$ *w.r.t.* the new contracted graph $G_c \oplus \Delta G_c$.

$\mathsf {ICP}$ studies the maintenance of contracted graphs in response to update $\Delta G$ that may both change the topological structures of contracted graph $G_c$, and refresh timestamps of nodes. As a consequence, obsolete nodes may be promoted to be non-obsolete ones if they are touched by $\Delta G$, among other things.

* Criterion*. Following [77], we measure the complexity of incremental algorithms with the size of the *affected area*, denoted by $\mathsf {AFF}$. Here $\mathsf {AFF}$ includes (a) changes $\Delta G$ to the input, (b) changes $\Delta G_c$ to the output, and (c) edges with at least an endpoint in (a) or (b).

An incremental algorithm is said to be *bounded* [77] if its complexity is determined by $|{\mathsf {AFF}} |$, not by the size |*G*| of the entire (possibly big) graph *G*.

Intuitively, $\Delta G$ is typically small in practice. When $\Delta G$ is small, so is $\Delta G_c$. Hence, when $\Delta G$ is small, a bounded incremental algorithm is often far more efficient than a batch algorithm that recomputes $G_c$ starting from scratch, since the cost of the latter depends on the size of *G*, as opposed to $|{\mathsf {AFF}} |$ of the former.

An incremental problem is said to be *bounded* if there exists a bounded incremental algorithm for it, and it is *unbounded* otherwise.

* Challenges*. Problem $\mathsf {ICP}$ is nontrivial. (1) Topological components are fragile. For instance, when inserting an edge between two leaves of a star *H*, *H* is no longer a star, and its nodes may need to be merged into other topological components. (2) Refreshing timestamps by a query *Q* may make some obsolete nodes “fresh” and force us to reorganize obsolete and topological components. (3) When contracted graph $G_c$ is changed, so are their associated synopses and decontraction function.

*Main result*. Despite challenges, we show that bounded incremental contraction is within reach in practice.

#### Theorem 2

Problem $\mathsf {ICP}$ is bounded for $\mathsf {SubIso}$, $\mathsf {TriC}$, $\mathsf {Dist}$, $\mathsf {CC}$ and $\mathsf {CD}$, and takes at most $O(|{\mathsf {AFF}} |^2)$ time.

We first give a constructive proof of Theorem 2 for edge updates, consisting of two parts: (1) the maintenance of the contracted graph $G_c$ and its associated decontraction function $f_D$ (Sect. 4.2); and (2) the maintenance of the synopses of affected supernodes (Sect. 4.3). We then give a constructive proof of Theorem 2 for vertex updates (Sect. 4.4), which is simpler.

### Incremental contraction algorithm

**Fig. 7:**
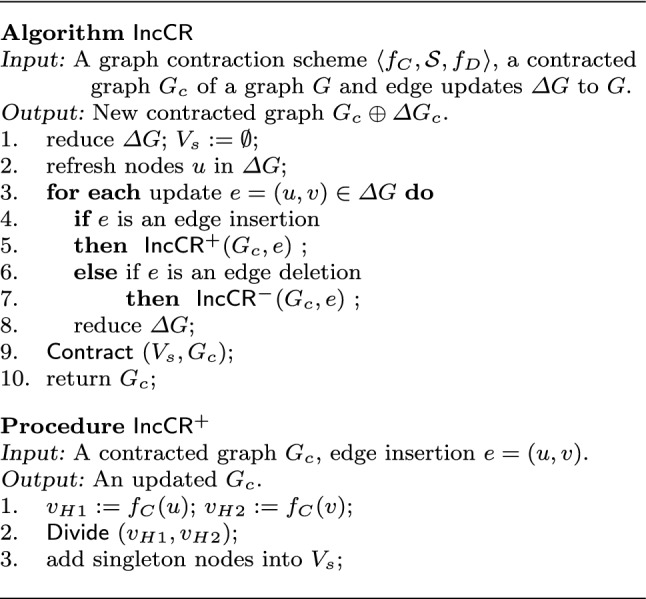
Algorithm $${\mathsf {IncCR}} $$

An incremental algorithm is shown in Fig. 7, denoted by $\mathsf {IncCR}$. It has three steps: *preprocessing* to initialize affected areas, *updating* to maintain contracted graph $G_c$, and *contracting* to process refreshed singleton nodes. To simplify the discussion, we focus on how to update $G_c$ in response to $\Delta G$, where $\Delta G$ consists of edge insertions and deletions; the handling of $f_D$ is similar.

* (a) Preprocessing*. Algorithm $\mathsf {IncCR}$ first identifies an initial area affected by edge update $\Delta G$ (lines 1-2). It removes “unaffecting” updates from $\Delta G$ that have no impact on $G_c$ (line 1), *i.e.,*  edges in $\Delta G$ that are between two supernodes when none of their nodes is an intermediate node of a path. These updates are made to corresponding subgraphs of *G* that are maintained by $f_D$. It then refreshes timestamps of nodes *u* touched by edges $e=(u,v)$ in $\Delta G$ (line 2). Suppose that node *u* is mapped by $f_C$ to supernode $v_H$ with $v_H.{\mathsf {type}}$ = obsolete. Then, $v_H$ is decomposed into singleton nodes, *u* is non-obsolete and is mapped to itself by $f_C$. Such singleton nodes are collected in a set $V_s$, as the initial area affected by $\Delta G$. Node *v* is treated similarly.

Note that an unaffecting update would not become “affecting update” later on. All changes in $\Delta G$ are applied to graph *G* in the given order.

* (b) Updating*. Algorithm $\mathsf {IncCR}$ then updates contracted graph $G_c$ (lines 3-8). For each update $e=(u,v)$, $\mathsf {IncCR}$ invokes procedure $\mathsf {IncCR^+}$ (resp. $\mathsf {IncCR^-}$) to update $G_c$ when *e* is to be inserted (resp. deleted) (lines 4-7). Updating $G_c$ may make some updates in $\Delta G$ unaffecting, which are further removed from $\Delta G$ (line 8). Moreover, some nodes may become “singleton” when a topological component is decomposed by the updates, *e.g.,* leaves of a star. It collects such nodes in the set $V_s$.

More specifically, to insert an edge $e=(u,v)$, $\mathsf {IncCR^+}$ updates $G_c$ and adds new singleton nodes to $V_s$. Suppose that *u* (resp. *v*) is mapped by $f_C$ to supernode $v_{H1}$ (resp. $v_{H2}$) (line 1). $\mathsf {IncCR^+}$ decomposes $v_{H1}$ and $v_{H2}$ into the regular structures of topological components (line 2). For instance, if $v_{H1}=v_{H2}$, and $v_{H1}.{\mathsf {type}} =$star, *u* and *v* make a triangle with the central node; thus, $\mathsf {IncCR^+}$ decomposes the star into singleton nodes. When $v_{H1}.{\mathsf {type}} ={\mathsf {clique}}$ and $v_{H2}.{\mathsf {type}} ={\mathsf {path}}$, supernode $v_{H_2}$ is divided into two shorter paths. Note that components with less than $k_l$ nodes due to updates are decomposed into singleton nodes. All such singleton nodes are added to the set $V_s$ (line 3).

* (c) Contracting*. Finally, algorithm $\mathsf {IncCR}$ processes nodes in the set $V_s$ (line 10). It (a) merges nodes into neighboring supernodes; or (b) builds new components with these nodes, if possible; otherwise (c) it leaves node *v* as a singleton, *i.e.,* by letting $f_C(v) = v$.

#### Example 11

Consider inserting four edges into graph *G* of Fig. 1a: (1) $(n_1, f_1)$: nodes $n_1$ and $f_1$ are mapped to obsolete component $v_{H1}$, and $v_{H1}$ is decomposed into singleton nodes, one for each of $n_1$, $f_1$, $i_1$ and $l_1$; then, $(n_1, f_1)$ is removed from $\Delta G$; (2) $(k_1, u_4)$: it is unaffecting since $f_C(k_1) \ne f_C(u_4)$ and neither $k_1$ nor $u_4$ is an intermediate node of a path; (3) $(k_1, u_{10})$: it is also unaffecting; and (4) $(u_1, u_4)$: $v_{H4}$ is not a butterfly any longer, and is decomposed into singletons.

Edge deletions are handled similarly. $\square$

* Analyses*. Algorithm $\mathsf {IncCR}$ takes $O(|{\mathsf {AFF}} |^2)$ time: (a) the preprocessing step is in $O(|\Delta G|)$ time; (b) the updating step takes $O(|{\mathsf {AFF}} |)$ time, in which updating $f_D$ is the dominating part; and (3) the cost of contracting $V_s$ into topological components is in $O(|{\mathsf {AFF}} |^2)$ time.

The algorithm is (a) bounded [77], since its cost is determined by $|{\mathsf {AFF}} |$ alone, and (b) *local* [35], *i.e.,* the changes are confined only to affected supernodes and their neighbors in the contracted graph $G_c$.

### Maintenance of synopses

We next show that for $\mathsf {SubIso}$, $\mathsf {TriC}$, $\mathsf {Dist}$, $\mathsf {CC}$ and $\mathsf {CD}$, (a) the number of supernodes whose synopses are affected is at most $O(|{\mathsf {AFF}} |)$, and (2) the synopsis for each supernode can be updated in $O(|{\mathsf {AFF}} |)$ time. Hence, incremental synopses maintenance for each of $\mathsf {SubIso}$, $\mathsf {TriC}$, $\mathsf {Dist}$, $\mathsf {CC}$ and $\mathsf {CD}$ takes at most $O(|{\mathsf {AFF}} |^2)$ time.

To see these, consider a supernode $v_H$ in $G_c$.

(a) For $\mathsf {SubIso}$, recall that $S_{\mathsf {SubIso}} (v_H)$ stores the type and key features of $v_H$ (Sect. 3.1). One can see that the number of supernodes whose synopses are affected is at most $|\Delta G_c|$, and $S_{\mathsf {SubIso}} (v_H)$ for each such $v_H$ can be updated in *O*(1) time. Thus, the maintenance of $S_{\mathsf {SubIso}}$ is bounded in $O(|{\mathsf {AFF}} |)$ time due to bounds $[k_l, k_u]$.

(b) For $\mathsf {TriC}$, synopsis $S_{\mathsf {TriC}} (v_H)$ extends $S_{\mathsf {SubIso}} (v_H)$ with $v_H.{\mathsf {tc}}$, which is updated by (i) clique neighbors *I* of nodes *u* in $v_H$ when $I \in {\mathsf {AFF}}$; (ii) $v_H$ itself if $v_H.{\mathsf {type}}$ is clique or obsolete; and (iii) common neighbors *J* of connected nodes *u*, *v* in $v_H$ for $J \in {\mathsf {AFF}}$. Thus, supernodes affected are enclosed in ${\mathsf {AFF}}$, which covers $\Delta G$, $\Delta G_c$ and their neighbors. Moreover, $S_{\mathsf {TriC}} (v_H)$ for each affected $v_H$ can be updated in $|{\mathsf {AFF}} |$ time. Thus, the maintenance of $S_{\mathsf {TriC}}$ is bounded in $O(|{\mathsf {AFF}} |^2)$ time.

(c) For $\mathsf {Dist}$, $S_{\mathsf {Dist}} (v_H)$ extends $S_{\mathsf {SubIso}} (v_H)$ with $v_H.{\mathsf {dis}}$, which is confined to $v_H$ and can be updated in *O*(1) time since $|f_C'(v_H)| \le k_u$. Thus, the incremental maintenance of $S_{\mathsf {Dist}}$ is bounded in $O(|{\mathsf {AFF}} |)$ time.

(d) For $\mathsf {CC}$, recall that the synopsis $S_{\mathsf {SubIso}}$ suffices to answer $\mathsf {CC}$ queries. Hence, as in case (a), $S_{\mathsf {CC}} (v_H)$ for each supernode $v_H$ can be updated in *O*(1) time, and the maintenance of $S_{\mathsf {CC}}$ is bounded in $O(|{\mathsf {AFF}} |)$ time.

(e) For $\mathsf {CD}$, $S_{\mathsf {CD}} (v_H)$ extends $S_{\mathsf {SubIso}} (v_H)$ with $v_H.{\mathsf {cs}}$ and $v_H.{\mathsf {md}}$. Here $v_H.{\mathsf {cs}}$ is confined to $v_H$ and can be updated in *O*(1) time; $v_H.{\mathsf {md}}$ is confined to $v_H$ and its neighbors, and can be updated in $O(|{\mathsf {AFF}} |)$ time. Thus, the maintenance of $S_{\mathsf {CD}}$ is in $O(|{\mathsf {AFF}} |^2)$ time.

#### Example 12

Continuing with Example 11, we show how to maintain $v_H.{\mathsf {tc}}$ in $S_{\mathsf {TriC}} (v_H)$ for supernodes $v_H$ in $G_c$; $S_{\mathsf {SubIso}} (v_H)$, $S_{\mathsf {Dist}} (v_H)$, $S_{\mathsf {CC}} (v_H)$ and $S_{\mathsf {CD}} (v_H)$ are simpler since their affected synopses are confined to $\Delta G_c$.

More specifically, (1) for edge insertion $(n_1,f_1)$, supernode $v_{H1}$ is decomposed into four singletons, for which synopses are defined as $n_1.{\mathsf {tc}} =f_1.{\mathsf {tc}} =l_1.{\mathsf {tc}} =i_1.{\mathsf {tc}} =0$. (2) For (unaffecting) edge insertion $(k_1, u_4)$, $v_H.{\mathsf {tc}}$ remains the same for all $v_H \in G_c$. (3) For (unaffecting) edge insertion $(k_1, u_{10})$, $k_1$ becomes a common neighbor of $u_{10}$ and $u_6$; let *H* denote the subgraph contracted by $v_{H2}$; then, $t_{u_{10},u_6}^{H}=1$ and $v_{H3}.{\mathsf {tc}} =1$. (4) When inserting edge $(u_1, u_4)$, $v_{H4}$ is decomposed into singletons. During the contraction phase, nodes $u_1,u_2,u_5,u_4$ are contracted into a diamond $v_{H4}'$ with $v_{H4}'.{\mathsf {tc}} =2$. Node $u_3$ is left singleton, with $u_3.{\mathsf {tc}} =0$. $\square$

### Vertex updates

**Fig. 8:**
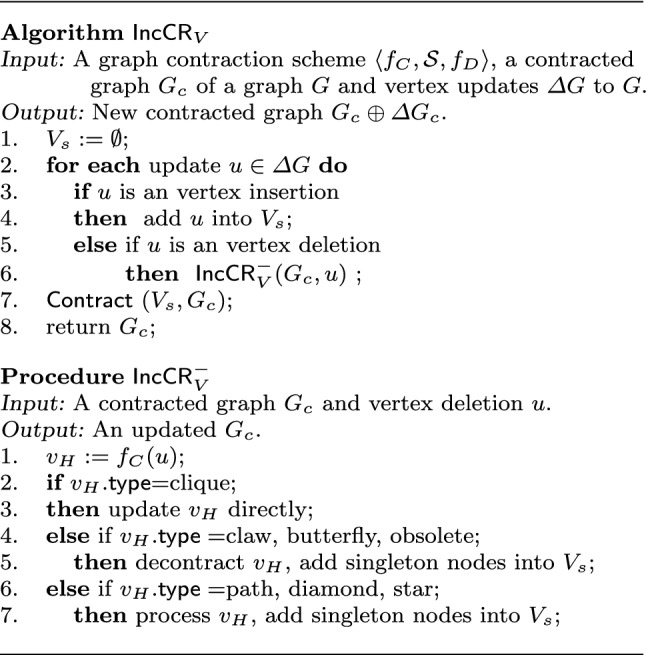
Algorithm $${\mathsf {IncCR}} _V$$

Vertex updates are a dual of edge updates [58], and can be processed accordingly. More specifically, we present incremental algorithm ${\mathsf {IncCR}} _V$ in Fig. 8, to deal with vertex updates. Consider node insertions and deletions.

(1) When inserting a new node *u*, algorithm ${\mathsf {IncCR}} _V$ first treats *u* as a singleton and collects it in set $V_s$ (lines 3-4); the node *u* is then contracted into a topological structure in the contracting step (line 7).

(2) When deleting a node *u* that is contracted into a supernode $v_H$, there are three cases to consider, elaborated in ${\mathsf {IncCR}} _V^-$ of Fig. 8: (a) if $v_H$ is a clique, $v_H$ remains unchanged except that *u* is removed (lines 2-3); (b) if $v_H$ is a claw, a butterfly or an obsolete component, $v_H$ is decontracted and all nodes in $f_C'(v_H)$ except *u* are treated as singletons and are collected in set $V_s$ (lines 4-5); and (c) otherwise, we process *u* and $v_H$ by synopsis and add resulting singleton nodes into $V_s$ (lines 6-7). For instance, consider the case when $v_H$ contracts a star, (i) if *u* is the central node $v_H.c$, $v_H$ is decontracted in the same way as case (b); and (ii) otherwise, $v_H$ remains to be a star, similar to case (a).

Similar to edge updates, contracting singleton nodes of $V_s$ into topological components dominates the cost of the process. One can verify that it can be done in at most $O(|{\mathsf {AFF}} |^2)$ time. Similarly, synopsis maintenance also takes $O(|{\mathsf {AFF}} |^2)$ time. Hence, incremental contraction remains bounded in the presence of vertex updates.

### Parallel incremental contraction algorithm

We parallelize incremental algorithm $\mathsf {IncCR}$, to speed up the incremental maintenance process.

**Parallel setting**. Similar to $\mathsf {PCon}$, we use a master $M_0$ and *n* workers, A contracted graph $G_c$ is edge-partitioned and is distributed to *n* workers. Each fragment $F_i$ consists of a part of the contracted graph $G_c$ and its corresponding (partial) decontraction function and synopses. For a *crossing superedge* $(v_{H1}, v_{H2})$ between two fragments, *i.e.,* when $v_{H1}$ and $v_{H2}$ are assigned to two distinct fragments, the decontraction function $f_D(v_{H1},v_{H2})$ is maintained in both fragments.

**Fig. 9:**
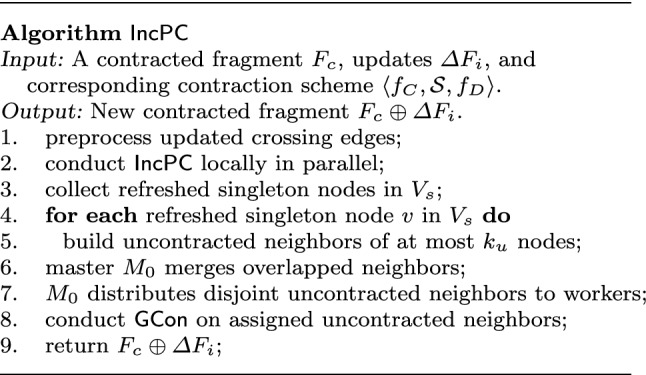
Algorithm $${\mathsf {IncPC}} $$

**Parallel incremental contraction**. The parallel incremental algorithm is denoted by $\mathsf {IncPC}$ and shown in Fig. 9. To simplify the discussion, we focus on edge updates; node updates are processed similarly. It works under $\mathsf {BSP}$ [88]. In a nutshell, it preprocesses crossing (super)edges (line 1). Then, all the workers run $\mathsf {IncCR}$ on its local fragment in parallel (line 2). After that, $\mathsf {IncPC}$ contracts refreshed singleton nodes $V_s$ into supernodes (lines 3-8) along the same lines as algorithm $\mathsf {PCon}$. Here, each fragment has its local set $V_s$ and all refreshed singleton nodes in $V_s$ can be coordinated and distributed by the master $M_0$. Each node *v* is guaranteed to be contracted into one supernode $v_H$. More specifically, algorithm $\mathsf {IncPC}$ works as follows.

(1) $\mathsf {IncPC}$ preprocess updated edges $e = (u,v)$ between two fragments (line 1), *i.e.,* when *u* and *v* are contracted into supernodes $v_{H1}$ and $v_{H2}$, and $v_{H1}$ and $v_{H2}$ are in two distinct fragments. Such updates are unaffecting as long as neither *u* nor *v* is an intermediate node of a path, and these updates are maintained by $f_D$. Otherwise, the supernode of type *path* may be affected and is decomposed into singleton nodes; such refreshed singleton nodes are collected in a set $V_s$ as the initial area affected by $\Delta G$. In the same way as $\mathsf {IncCR}$, we refresh timestamps of obsolete nodes touched by updates.

(2) Each worker locally runs $\mathsf {IncCR}$ in parallel (line 2). Refreshed singleton nodes that cannot be contracted into supernodes are collected in $V_s$ (line 3).

(3) For each refreshed singleton node *v* in $V_s$, $\mathsf {IncPC}$ build its uncontracted neighbors (of at most $k_u$ nodes) in parallel, similar to step (2) in $\mathsf {PCon}$ (lines 4-5).

(4) Master $M_0$ merges overlapped neighbors into one and distributes disjoint ones to *n* workers (lines 6-7).

(5) Each worker contracts its assigned subgraphs, *i.e.,* uncontracted neighbors, in parallel (line 8).

One can verify that each node *v* in *G* is contracted into one supernode $v_H$ (including *v* itself), and the contracted graph $G_c$ cannot be further contracted.

**Table 2:** Contraction ratio (each column: *CR* or % of contribution to *CR* with/without obsolete mark)

Graph	|*V*|, |*E*|	$k_u$	*CR*	1st	2nd	3rd	Obsolete
Twitter	81K, 1.3M	100	0.176/0.286	7.78/27.7	15.44/50.71	4.29/14.39	69.69/–
LiveJournal	4M, 35M	500	0.378/0.527	11.46/30.3	20.41/51.4	3.74/9.7	60.99/–
LivePokec	1.6M, 22M	500	0.467/0.651	4.46/9.91	35.91/77.76	2.32/4.83	54.4/–
Google	876K, 4.3M	200	0.193/0.294	19.36/51.47	19.33/47.04	0.58/1.49	60.74/–
NotreDame	325K,1.1M	200	0.274/0.441	23.16/60.64	9.47/26.95	4.56/12.4	62.81/–
GSH	68M, 1.8B	500	0.325/0.493	29.32/77.33	5.31/21.78	0.75/0.89	64.62/–
DBLP	204K, 382K	100	0.14/0.172	36.21/71.65	14.22/28.32	0.02/0.03	49.54/–
Hollywood	1.1M, 56M	500	0.239/0.534	17.36/71.76	6.05/16.46	3.21/11.79	73.38/–
citHepTh	28K, 352K	50	0.26/0.362	21.42/51.93	14.18/36.71	4.6/11.36	59.81/–
Traffic	24M, 29M	500	0.365/0.59	12.37/49.72	9.42/36.74	3.5/13.54	74.7/–

## Experimental study

Using ten real-life graphs, we experimentally evaluated (1) the contraction ratio; (2) the speedup of the contraction scheme; (3) the impact of contracting each topological component and obsolete component; (4) the space cost of the contraction scheme compared to existing indexing methods; (5) the efficiency of the (incremental) contraction algorithms; and (6) the parallel scalability of the (incremental) contraction algorithms.

**Experiment setting**. We used the following datasets.

* (1) Graphs*. We used ten real-life graphs: three social networks $\mathsf {Twitter}$ [70], $\mathsf {LiveJournal}$ [94] and $\mathsf {LivePokec}$ [10]; three Web graphs $\mathsf {Google}$ [64], $\mathsf {NotreDame}$ [5] and $\mathsf {GSH}$ [3]; three collaboration networks $\mathsf {DBLP}$ [2], $\mathsf {Hollywood}$ [15] and $\mathsf {citHepTh}$ [63]; and a road network $\mathsf {Traffic}$ [1]. Their sizes are shown in Table 2. We randomly generated a time series to simulate obsolete attributes, at most 70% (it is 80% for IT data of our industry collaborator). We tested obsolete components with random (temporal) queries generated on all datasets.

We also generated synthetic graphs with up to 250 M nodes and 2.5 B edges, to test the parallel scalability of the (incremental) contraction algorithms.

* Updates*. We randomly generated edge updates $\Delta G$, controlled by the size $|\Delta G|$ and a ratio $\rho$ of edge insertions to deletions. We kept $\rho = 1$ unless stated otherwise, *i.e.,* the size of $G \oplus \Delta G$ remains stable. In the same manner, we generated vertex updates $\Delta G$.

* (2) Graph patterns*. We implemented a generator for graph pattern queries controlled by three parameters: the number $V_Q$ of pattern nodes, the number $E_Q$ of pattern edges, and a set $L_Q$ of labels for queries *Q*.

* (3) Implementation.* We implemented the following algorithms, all in C++. (1) Algorithms $\mathsf {SubA_{c}}$ (Sect. 3.1.2), $\mathsf {TriA_{c}}$ (Sect. 3.2.2), $\mathsf {DisA_{c}}$ (Sect. 3.3.2), $\mathsf {CCA_{c}}$ (Sect. 3.4.2), $\mathsf {CDA_{c}}$ (Sect. 3.5.2), $\mathsf {VF2_{c}}$ for $\mathsf {SubIso}$ by adapting $\mathsf {VF2}$ [28] to contracted graphs; in addition, $\mathsf {PLL_{c}}$ for $\mathsf {Dist}$ by adapting $\mathsf {PLL}$ [4] to contracted graphs. (2) Our contraction algorithm $\mathsf {GCon}$ (Sect. 2.3) and its parallel version $\mathsf {PCon}$ (Sect. 2.4), incremental algorithm $\mathsf {IncCR}$ for batch updates and its parallel version $\mathsf {IncPC}$ (Sect. 4). (3) The baselines include existing query evaluation algorithms: (a) $\mathsf {TurboIso}$ [44] and $\mathsf {TurboIsoBoosted}$ [78] with indexing for $\mathsf {SubIso}$, and $\mathsf {VF2}$ [28] without indexing; (b) graph compression $\mathsf {DeDense}$ [69] for $\mathsf {SubIso}$; (c) $\mathsf {TriA}$ [47] for $\mathsf {TriC}$; (d) $\mathsf {Dijkstra}$ without indexing and $\mathsf {PLL}$ [4] with indexing for $\mathsf {Dist}$ [31]; (e) $\mathsf {CCA}$ [85] for $\mathsf {CC}$; and (f) $\mathsf {CDA}$ [57] for $\mathsf {CD}$. We did not compare with summarization since it does not support any algorithm to compute exact answers for the five applications.

* (4) Experimental environment*. The experiments were conducted on a single-processor machine powered by Xeon 3.0 GHz with 64GB memory, running Linux. Since $\mathsf {GSH}$ and synthetic graphs ran out of 32 GB memory without contraction, we used a machine with 64 GB memory. For parallel (incremental) contraction, we used 4 machines, each with 12 cores powered by Xeon 3.0 GHz, 32GB RAM, and 10Gbps NIC. Each experiment was run 5 times, and the average is reported here.

**Experimental results**. We now report our findings.

**Exp-1: Effectiveness: Contraction ratio**. We first tested the *contraction ratio* of our contraction scheme, defined as $CR = |G_c|/|G|$. Note that for each query class $\mathcal {Q}$, *CR* is the same for all queries in $\mathcal {Q}$. Moreover, all applications on *G* share the same contracted graph $G_c$ while incorporating different synopses. In addition, we report the impact of each of the first three topological components and obsolete component for each dataset, in the presence and absence of obsolete data.

As remarked in Sect. 2, we limit the nodes of contracted subgraphs within $[k_l, k_u]$. We fixed $k_l = 4$ and varied $k_u$ based on the size of each graph. We considered two settings: (a) when obsolete data are taken into account, with threshold $t_0 = 50\%t_m$, where $t_m$ denotes the maximum timestamp in each dataset; and (b) when we do not separate obsolete data, *i.e.,* when $t_0 = 0$. The results are reported in Table 2 for all the real-life graphs (in which each column indicates either *CR* or percentage of contribution to *CR* with/without obsolete mark). We can see the following.

(1) When $t_0 = 50\%t_m$, *CR* is on average 0.281, *i.e.,*  contraction reduces these graphs by 71.9%. When $t_0 = 0$, *i.e.,* if obsolete data are not considered, *CR* is 0.435. These show that real-life graphs can be effectively contracted in the presence and absence of obsolete data. Compared with the results of [38], by considering more regular structures, the contraction scheme improves the contraction ratio *CR* by 2.49% and 6.90% in the presence and absence of obsolete data, respectively.

(2) When obsolete data are present, the average *CR* is 0.34, 0.264, 0.213 and 0.365 in social networks, Web graphs, collaboration networks and road networks, respectively. When obsolete data are absent, *CR* is on average 0.488, 0.409, 0.356 and 0.59. The contraction scheme performs the best on collaboration networks in both settings, since such graphs exhibit evident inhomogeneities and community structures.

**Table 3:** Slowdown (%) by RE and EX orders

**Graph**	$\mathsf {SubIso}$		$\mathsf {TriC}$		$\mathsf {Dist}$		$\mathsf {CC}$		$\mathsf {CD}$	
	RE	EX	RE	EX	RE	EX	RE	EX	RE	EX
Twitter	8.04	3.98	7.41	3.66	5.27	2.86	8.22	19.2	6.73	24.9
LiveJournal	9.46	5.52	8.26	5.09	2.71	5.32	9.03	61.1	5.49	18.3
LivePokec	8.67	6.46	3.15	3.12	4.49	2.48	6.10	51.7	8.23	20.2
Google	5.17	7.54	6.07	3.75	1.02	3.8	7.19	38.7	$-4.17$	12.5
NotreDame	11.9	5.76	4.20	6.46	5.95	4.93	3.72	44.5	$-4.33$	15.3
GSH	3.52	6.22	4.59	6.08	2.78	4.15	4.25	32.1	$-5.53$	16.4
DBLP	2.13	5.53	11.3	14.2	4.38	5.31	18.8	19.6	5.05	34.2
Hollywood	6.32	6.39	2.25	4.73	3.89	5.81	5.75	30.3	3.02	29.3
citHepTh	7.48	3.24	3.98	4.91	2.56	3.23	7.43	35.5	7.92	17.1
Traffic	9.69	5.11	4.29	2.56	5.78	5.11	2.94	14.2	1.39	2.87

(3) When obsolete data are absent, on average the first three regular structures contribute 50.2%, 39.4% and 8.0% to CR, respectively. When obsolete mark is taken into account, their contribution is 18.3%, 14.9% and 2.8%, respectively. This is because nodes from these components may be moved to obsolete components.

(4) We also studied the impact of the contraction order on query evaluation. Topological components have different impacts on different types of graphs, *e.g.,* stars, claws and paths are effective in $\mathsf {Traffic}$, and cliques, stars and butterflies work better than the others in collaboration networks. Taking the order of Table 1 as the baseline, we tested the impact of (a) RE, by reversing the order, and (b) EX, by exchanging between different types of graphs, *e.g.,* we use the order for road networks to contract social graphs. On average the CR of RE and EX is decreased by 9.42% and 7.05%, respectively. As shown in Table 3, the average slowdown of RE and EX is (a) 7.24% and 5.58% for $\mathsf {SubIso}$, (b) 5.55% and 5.46% for $\mathsf {TriC}$, (c) 3.89% and 4.30% for $\mathsf {Dist}$, (d) 7.34% and 34.7% for $\mathsf {CC}$, and (e) 2.38% and 19.1% for $\mathsf {CD}$, respectively. These justify that the order of Table 1 is effective for most applications and most types of graphs. There are also exceptions, *e.g.,* reversing the order for Web graphs improves the efficiency of $\mathsf {CD}$. Recall that we contract stars, cliques and butterflies for Web graphs. For $\mathsf {CD}$ in particular, however, cliques play a more important role than the other two (Sect. 3.5); hence, contracting cliques first may work better for $\mathsf {CD}$.

**Fig. 10:**
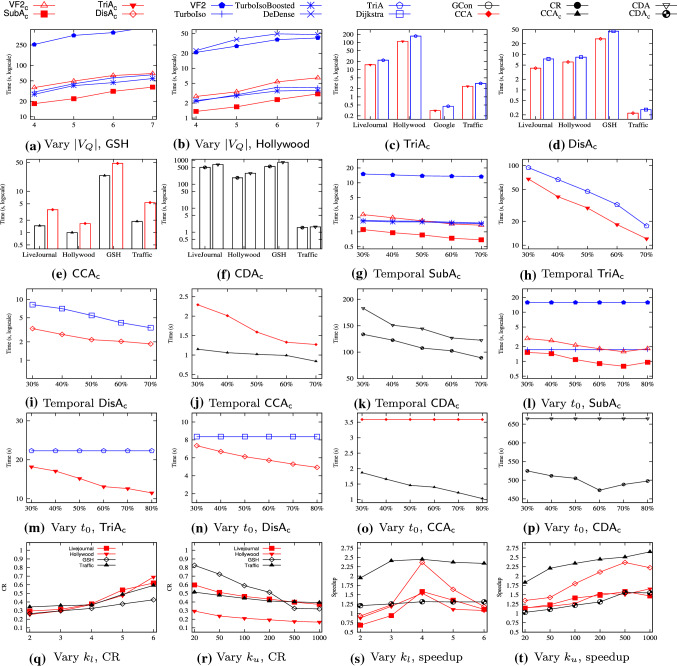
Performance evaluation

**Table 4:** Slowdown(%) by disabling certain topological component

Graph	$\mathsf {SubIso}$			$\mathsf {TriC}$			$\mathsf {Dist}$			$\mathsf {CC}$			$\mathsf {CD}$		
	1st	2nd	3rd	1st	2nd	3rd	1st	2nd	3rd	1st	2nd	3rd	1st	2nd	3rd
Twitter	45.8	10.9	4.7	16.4	19.1	2.1	28.2	28.7	5.7	85.1	141.6	22.8	42.7	4.1	0.3
LiveJournal	46.3	16.7	3.0	17.5	3.9	1.4	44.3	13.2	7.1	68.4	95.5	14.5	27.5	3.3	0.9
LivePokec	45.5	13.5	2.1	5.5	22.1	0.7	29.5	23.6	4.4	18.0	69.5	11.3	39.0	5.2	1.3
GSH	11.7	32.2	0.4	5.4	18.2	1.1	15.9	33.1	0.7	41.7	10.8	0.4	4.9	52.2	0.2
Google	19.6	40.6	2.5	8.7	20.3	2.9	18.3	44.6	5.8	107.1	70.8	5.6	5.4	57.8	2.7
NotreDame	15.2	42.3	3.3	29.5	41.2	0.4	27.7	47.8	4.9	55.4	50.2	8.0	2.1	20.6	0.5
DBLP	66.6	17.0	0.8	572.1	216.6	1.7	23.2	29.5	0.4	631.7	450.2	0.1	65.1	7.9	0.1
Hollywood	40.3	13.4	5.1	22.6	10.9	1.5	24.0	26.3	5.4	80.1	64.3	5.9	51.7	3.8	0.2
citHepTh	54.5	15.7	2.4	15.4	7.2	0.5	32.3	22.6	7.3	280.7	222.4	25.7	35.5	1.7	0.6
Traffic	30.1	24.3	5.7	10.1	3.5	9.4	40.2	18.7	10.6	41.7	8.7	5.2	4.3	2.5	0.3

**Exp-2: Effectiveness: query processing**. We next evaluated the speedup of query processing introduced by the contraction scheme, measured by query evaluation time over original and contracted graphs.

* Subgraph isomorphism*. Varying the size $|V_Q|$ of pattern queries from 4 to 7, we tested $\mathsf {VF2}$, $\mathsf {TurboIso}$ and $\mathsf {TurboIsoBoosted}$ on $\mathsf {GSH}$ and $\mathsf {Hollywood}$ as *G*, $\mathsf {DeDense}$ [69] on the compressed graph, and $\mathsf {SubA_{c}}$ and $\mathsf {VF2_{c}}$ on the contracted graph $G_c$ of *G*. For each query, we output the first $10^8$ matches. As shown in Fig. 10a, b, (1) on average, $\mathsf {SubA_{c}}$ on $G_c$ is1.69, 1.49 and 18.85 times faster than $\mathsf {TurboIso}$, $\mathsf {TurboIsoBoosted}$ and $\mathsf {DeDense}$, respectively; (2) $\mathsf {VF2_{c}}$ beats $\mathsf {DeDense}$ by 9.31 times; (3) $\mathsf {VF2_{c}}$ without indices is only 19.1% slower than $\mathsf {TurboIso}$ with indices, while $\mathsf {TurboIsoBoosted}$ and $\mathsf {TurboIso}$ are 10.1 and 8.97 times faster than $\mathsf {VF2}$, respectively; and (4) the speedup is more substantial on collaboration networks, *e.g.,* 2.11 times on $\mathsf {Hollywood}$, because cliques are prevalent in such graphs and are the most effective structure for $\mathsf {SubIso}$ due to the high capacity in pruning invalid matches.

*Triangle counting*. As shown in Fig. 10c, the results for $\mathsf {TriC}$ are consistent with the results on subgraph isomorphism: (1) $\mathsf {TriA_{c}}$ on the contracted $G_c$ is on average 1.44 times faster than $\mathsf {TriA}$ on their original graphs *G*. (2) The speedup is more evident in collaboration networks: *e.g.,* $\mathsf {TriA_{c}}$ on $\mathsf {Hollywood}$ is 1.57 times faster than $\mathsf {TriA}$ while it is 1.47, 1.45 and 1.28 times on $\mathsf {LiveJournal}$, $\mathsf {Google}$ and $\mathsf {Traffic}$, respectively. $\mathsf {TriA}$ spends more than 1000 seconds on $\mathsf {GSH}$ (hence not shown).

* Shortest distance*. The results for $\mathsf {Dist}$ are consistent with the results on $\mathsf {SubIso}$. As reported in Fig. 10d, $\mathsf {DisA_{c}}$ is 1.64 and 1.36 times faster than $\mathsf {Dijkstra}$ on $\mathsf {GSH}$ and $\mathsf {Hollywood}$, respectively, by reducing search space and employing synopses. $\mathsf {PLL}$ could not build indices on $\mathsf {GSH}$ within 64G memory, while $\mathsf {PLL_{c}}$ successfully builds indices on (smaller) contracted $\mathsf {GSH}$. On average, $\mathsf {PLL_{c}}$ spends 94.2$\mu s$ to evaluate a query on $\mathsf {GSH}$. On other smaller datasets, in contrast, $\mathsf {PLL_{c}}$ is 18% slower than $\mathsf {PLL}$ due to overhead on supernodes.

* Connected component*. As shown in Fig. 10e over $\mathsf {LiveJournal}$, $\mathsf {GSH}$, $\mathsf {Hollywood}$, and $\mathsf {Traffic}$ for social graphs, Web graphs, collaboration networks and road networks, respectively, the results for $\mathsf {CC}$ are consistent with the results on $\mathsf {SubIso}$ and $\mathsf {TriC}$: (1) algorithm $\mathsf {CCA_{c}}$ on contracted graph $G_c$ is on average 2.24 times faster than $\mathsf {CCA}$ on the original graph *G*, since $\mathsf {CCA_{c}}$ operates on the smaller $G_c$ without decontracting supernodes or superedges. (2) The speedup is more evident in collaborations networks: *e.g.,* $\mathsf {CCA_{c}}$ on $\mathsf {Hollywood}$ is 2.87 times faster than $\mathsf {CCA}$, since the contraction scheme performs the best on such graphs and the time complexity of $\mathsf {CCA_{c}}$ is linear in the size of the contracted graph.

* Clique decision*. As also shown in Fig. 10f, (1) algorithm $\mathsf {CDA_{c}}$ is 1.32, 1.54, 1.52 and 1.08 times faster than $\mathsf {CDA}$ on $\mathsf {LiveJournal}$, $\mathsf {Hollywood}$, $\mathsf {GSH}$ and $\mathsf {Traffic}$, respectively, by using synopses to start with an initial maximum clique that may find a *k*-clique directly. (2) The speedup is less evident in road networks. For road networks, the contraction scheme contracts stars, claws and paths into supernodes; hence, we can only find a 2-clique (an edge) as the initial maximum clique by using synopses, which is trivial and useless.

The results on the other graphs are consistent.

* Temporal queries*. Fixing pattern size |*Q*| = 4 and varying timestamp *t* in temporal queries from $30\%t_m$ to $70\%t_m$, we tested ${\mathsf {SubIso}} _t$, ${\mathsf {TriC}} _t$, ${\mathsf {Dist}} _t$, ${\mathsf {CC}} _t$ and ${\mathsf {CD}} _t$. As shown in Fig. 10g–k on $\mathsf {LiveJournal}$, (1) $\mathsf {SubA_{c}}$  is on average 1.81 and 1.77 times faster than $\mathsf {TurboIsoBoosted}$ and $\mathsf {TurboIso}$, respectively; $\mathsf {VF2_{c}}$ outperforms $\mathsf {VF2}$ by 7.83 times. (2) The average speedup for $\mathsf {TriC}$, $\mathsf {Dist}$, $\mathsf {CC}$ and $\mathsf {CD}$ is 1.58, 2.31, 1.66 and 1.31 times, respectively. (3) The speedup is larger for temporal queries than for conventional ones since temporal information maintained in synopsis provides additional capacity to skip more supernodes, as expected. (4) It is more substantial for larger *t* on ${\mathsf {SubIso}} _t$.

The results verify that our contraction scheme (a) is generic and speeds up evaluation for all five applications, and (b) it can be used together with existing algorithms, with indexing (*e.g.,* $\mathsf {TurboIso}$ and $\mathsf {PLL}$) or not (*e.g.,* $\mathsf {VF2_{c}}$ and $\mathsf {Dijkstra}$). (c) It is effective by separating up-to-date data from obsolete.

We remark that our contraction scheme aims to make a generic optimization for multiple applications to run on the same graph at the same time. When a new application is considered, adding a specific synopsis suffices for our scheme. In contrast, a separate indexing structure has to be built for indexing approaches. Better still, it is much easier to develop synopses than indices. Moreover, existing indexing structures can be inherited by contracted graphs, to improve performance from contraction in addition to from indexing.

**Exp-3: Impact of each component**. We next evaluated the impact of contracting each of the topological components identified in Sect. 2.2.

* Impact of topological components*. Based on Table 1, we took contraction of the first three types of regular structures as the baseline, and tested the impact of each component on the efficiency of query answering by disabling it, using all the ten real-life datasets.

As shown in Table 4, the average slowdown in evaluation time by disabling each of the first three structures is (a) 37.6%, 22.7% and 3.02% for $\mathsf {SubIso}$, (b) 70.3%, 36.3% and 2.0% for $\mathsf {TriC}$, (c) 28.4%, 28.8% and 5.2% for $\mathsf {Dist}$, (d) 141.0%, 118.4% and 9.9% for $\mathsf {CC}$, and (e) 27.8%, 15.9% and 0.7% for $\mathsf {CD}$, respectively. In particular, the impact of each regular structure is mostly consistent with the contraction order. This said, for specific application and graphs, the impact of each regular structure may be slightly different. For $\mathsf {CD}$ on Web graphs, the average slowdown in evaluation time by disabling the first structure (star) and the second structure (clique) is 4.1% and 43.5%, respectively, since cliques dominate the effectiveness of the synopses for $\mathsf {CD}$.

* Impact of obsolete components*. We tested the impact of contracting obsolete components on the efficiency of answering conventional queries. Fixing |*Q*| = 4 and varying *x* for timestamp threshold such that $t_0 = x\%t_m$, Fig. 10i–p reports the results of $\mathsf {SubIso}$, $\mathsf {TriC}$, $\mathsf {Dist}$, $\mathsf {CC}$ and $\mathsf {CD}$ on $\mathsf {LiveJournal}$, respectively. We find that (1) the speedup is bigger for larger $t_0$ when $t_0 \le 70\%$, *i.e.,* more nodes are contracted into obsolete components; (2) obsolete components speed up $\mathsf {SubIso}$, $\mathsf {TriC}$, $\mathsf {Dist}$, $\mathsf {CC}$ and $\mathsf {CD}$ by 1.56, 1.53, 1.39, 2.49 and 1.33 times, respectively; and (3) the speedup for $\mathsf {SubIso}$ and $\mathsf {CD}$ gets smaller when $t_0 \ge 80\%$ due to the overhead of decontracting obsolete components. The results are consistent for $\mathsf {Dist}$, $\mathsf {TriC}$ and $\mathsf {CC}$, except that their speedup does not go down when $t_0$ gets larger since they do not need to decontract obsolete components.

* Impact of* $\underline{k_l}$ *and* $\underline{k_u}$. We also tested the impact of $k_l$ and $k_u$ on the contraction ratio CR and efficiency. As remarked in Sect. 2.3, diamonds, butterflies and claws have a fixed size, while cliques, stars and paths vary. Fixing $k_u = 500$ (resp. $k_l = 4$) and varying $k_l$ (resp. $k_u$) from 2 to 6 (resp. 20 to 1000), Fig. 10q (resp. Fig. 10r) reports the *CR* on $\mathsf {LiveJournal}$, $\mathsf {Hollywood}$, $\mathsf {GSH}$ and $\mathsf {Traffic}$, respectively. As shown there, CR decreases when $k_l$ decreases or $k_u$ increases. Similarly, Fig. 10s (resp. Fig. 10t) reports the speedup of $\mathsf {SubA_{c}}$, $\mathsf {TriA_{c}}$, $\mathsf {DisA_{c}}$, $\mathsf {CCA_{c}}$ and $\mathsf {CDA_{c}}$ on $\mathsf {Hollywood}$. Query evaluation is slowed down when $k_l \le 3$ or $k_u \ge 500$ for all algorithms except $\mathsf {CCA_{c}}$ and $\mathsf {TriA_{c}}$ due to excessive superedge decontractions or overlarge components. Recall that $\mathsf {CCA_{c}}$ decontracts neither supernodes nor superedges, and $\mathsf {TriA_{c}}$ precalculates triangles in both topological components and obsolete parts; hence, it prefers large $k_u$. We find that the best $k_l$ and $k_u$ for the datasets tested are around 4 and 500, respectively.

The results on the other graphs are consistent.

**Exp-4: Space cost**. We next studied the space cost of our contraction scheme compared with indexing cost. We consider six algorithms: $\mathsf {SubA_{c}}$, $\mathsf {TriA_{c}}$, $\mathsf {DisA_{c}}$, $\mathsf {CCA_{c}}$, $\mathsf {CDA_{c}}$ and $\mathsf {PLL_{c}}$. The space cost includes the sizes of the contracted graph $|G_c|$, decontraction function $|f_D|$ and the sizes of synopses; as shown in Sect. 3, $\mathsf {SubA_{c}}$, $\mathsf {TriA_{c}}$, $\mathsf {DisA_{c}}$, $\mathsf {CCA_{c}}$ and $\mathsf {CDA_{c}}$ do not need to decontract topological components; thus, we only uploaded $f_D$ for obsolete components and superedges into memory. In particular, $\mathsf {CCA_{c}}$ requires no decontraction (Theorem 1) and thus incurs no cost for storing $f_D$ at all. We compared the space cost with the indices used by $\mathsf {TurboIso}$, $\mathsf {HINDEX}$ [75], $\mathsf {PLL}$ [4] and $\mathsf {RMC}$ [68].

**Table 5:** Total space cost of applications run on $\mathsf {Google}$

Application	Contraction		Indexing	
	Detail	Space	Detail	Space
Shared parts	$G_c, f_D$	837MB	*G*	727MB
+$\mathsf {SubIso}$	${\mathcal {S}_{\mathsf {SubIso}}}$	848MB	$\mathsf {TurboIso}$	1.07GB
+$\mathsf {TriC}$	$\mathcal {S}_{\mathsf {TriC}}$	874MB	+$\mathsf {HINDEX}$	2.1GB
+$\mathsf {Dist}$	$\mathcal {S}_{\mathsf {Dist}}$	1.51GB	+$\mathsf {PLL}$	9.58GB
+$\mathsf {CC}$	–	1.51GB	–	9.58GB
+$\mathsf {CD}$	$\mathcal {S}_{\mathsf {CD}}$	1.62GB	+$\mathsf {RMC}$	12.9GB
+$\mathsf {kNN}$	$\mathcal {S}_{\mathsf {kNN}}$	1.75GB	+$\mathsf {Antipole}$	19.4GB

Table 5 shows how the space cost increases when more applications run on $\mathsf {Google}$ (*i.e.,* graph *G*). We find the following. (1) Our contraction scheme takes totally 1.62GB for $\mathsf {SubIso}$, $\mathsf {TriC}$, $\mathsf {Dist}$, $\mathsf {CC}$ and $\mathsf {CD}$, much smaller than 12.9GB taken by $\mathsf {TurboIso}$, $\mathsf {PLL}$, $\mathsf {HINDEX}$ and $\mathsf {RMC}$. (2) With the contraction scheme, graph *G* is no longer needed. That is, compared to *G*, the scheme uses 0.89GB additional space for the supernodes/edges in $G_c$ and synopses for all five applications. It trades affordable space for speedup. (3) Synopses $S_{\mathsf {SubIso}}$, $S_{\mathsf {TriC}}$, $S_{\mathsf {Dist}}$, $S_{\mathsf {CC}}$ and $S_{\mathsf {CD}}$ take 48.3% of the total space of contraction, *i.e.,* $G_c$ and $f_D$ dominate the space cost, which are shared by all applications. Hence, the more applications are supported, the more substantial the improvement in the contraction scheme is over indices.

To inherit the indexing structures of [44] and $\mathsf {PLL}$, we use 1.14GB additional space to build a compact index for $\mathsf {PLL_{c}}$ and on average 26MB for $\mathsf {SubA_{c}}$ on $\mathsf {Google}$. in addition to synopses $S_{\mathsf {Dist}}$ and $S_{\mathsf {SubIso}}$.

To verify the scalability with applications, we further adapted existing algorithms for k-nearest neighbors ($\mathsf {kNN}$) [92]. The total space cost of the scheme for the six applications is 1.75GB, *i.e.,* 18.1% increment for each. It accounts for only 9.0% of the indices for $\mathsf {TurboIso}$, $\mathsf {PLL}$, $\mathsf {HINDEX}$, $\mathsf {RMC}$ and $\mathsf {Antipole}$ [22] of $\mathsf {kNN}$.

**Exp-5: Efficiency of (incremental) contraction**. We next evaluated the efficiency of contraction algorithm $\mathsf {GCon}$ and incremental contraction algorithm $\mathsf {IncCR}$. We also studied the impact of the order and varied rates of updates on incremental $\mathsf {IncCR}$.

**Fig. 11:**
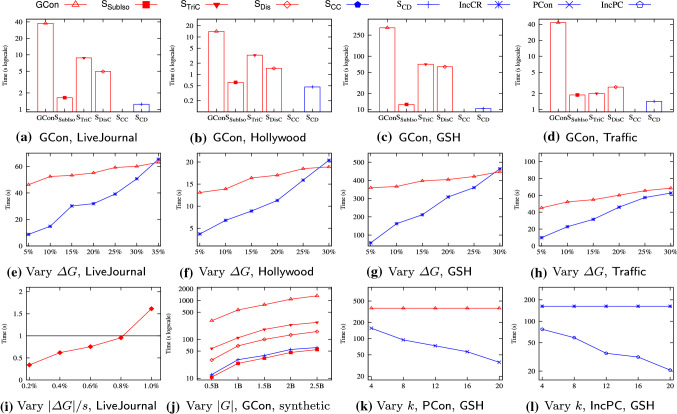
Efficiency of (incremental) contraction

* Efficiency of* $\underline{{\mathsf {GCon}}}$. We first report the efficiency of $\mathsf {GCon}$. As shown in Fig. 11a–d on $\mathsf {LiveJournal}$, $\mathsf {Hollywood}$, $\mathsf {GSH}$ and $\mathsf {Traffic}$, respectively, (1) on average $\mathsf {GCon}$ takes 109.7s to contract the graph, without the time of the computation for synopses. (2) It takes on average 4.13s, 21.2s, 18.1s, 0s and 3.38s only to compute the synopses for $\mathsf {SubIso}$, $\mathsf {TriC}$, $\mathsf {Dist}$, $\mathsf {CC}$ and $\mathsf {CD}$, respectively; *i.e.,* computing synopses of the five only takes on average 37.3% of the time of $\mathsf {GCon}$. Recall that the synopses for $\mathsf {SubIso}$ suffice for us to answer $\mathsf {CC}$ queries; hence, it is unnecessary to compute synopses for $\mathsf {CC}$.

* Efficiency of* $\underline{{\mathsf {IncCR}}}$. We tested the efficiency of $\mathsf {IncCR}$, by varying $|\Delta G|$ from $5\% |G|$ to $35\% |G|$. As shown in Fig. 11e–h on $\mathsf {LiveJournal}$, $\mathsf {Hollywood}$, $\mathsf {GSH}$ and $\mathsf {Traffic}$, respectively, (1) on average $\mathsf {IncCR}$ is 2.1 times faster than $\mathsf {GCon}$, up to 6.3 times when $|\Delta G| = 5\% |G|$. It takes on average 26.6% time to update the synopses for 5% updates on the five applications. (2) $\mathsf {IncCR}$ beats $\mathsf {GCon}$ even when $|\Delta G|$ is up to $30\% |G|$. This justifies the need for incremental contraction. (3) $\mathsf {IncCR}$ is sensitive to $|\Delta G|$; it takes longer for larger $|\Delta G|$.

* Impact of update order*. We tested the impact of the orders of edge insertions and deletions in $\Delta G$ on $\mathsf {IncCR}$. Fixing $|\Delta G| = 10\%$, we varied the order of updates by (1) random (RO), (2) insertion-first (IF) and (3) deletion-first (DF). On average we find that RO, IF and DF have a performance difference less than 3.5% on $\mathsf {Hollywood}$. That is, $\mathsf {IncCR}$ is *stable* on batch updates, regardless of the order on the updates. Similarly, we find that RO, IF and DF have a performance difference less than 3.7% on $\mathsf {Hollywood}$ for vertex updates.

* Impact of update rates*. We also tested the efficiency of $\mathsf {IncCR}$ against real-time updates, measured by the updates coming in 1s intervals, *i.e.,* $|\Delta G|$/s. Varying $|\Delta G|$/s from $0.2\% |G|$/s to $1\% |G|$/s, Fig. 11i shows the following on $\mathsf {LiveJournal}$. (1) On average it takes 0.88s to update contracted graphs, *i.e.,*  $\mathsf {IncCR}$ is able to efficiently maintain the contracted graphs in real life. (2) The update time is less than 1s even when the updates are up to $0.8\% |G|$. $\mathsf {IncCR}$ can handle $0.8\% |G|/s$ of “burst” updates on graph with 40M nodes and edges.

The results are consistent on the other graphs.

**Exp-6: Scalability**. Finally, we evaluated (1) the scalability of our contraction algorithm $\mathsf {GCon}$ with graph size |*G*|, (2) the parallel scalability of algorithm $\mathsf {PCon}$ and $\mathsf {IncPC}$ with the number of cores.

* Scalability on* $\underline{|G|}$. Varying the size $|G| = (|V|, |E|)$ of synthetic graphs from (50*M*, 0.5*B*) to (250*M*, 2.5*B*), we tested the scalability of $\mathsf {GCon}$  using a single machine. As shown in Fig. 11j, $\mathsf {GCon}$ scales well with *G*. It takes 1325s when graph *G* has 2.75*B* nodes and edges.

* Scalability of* $\underline{{\mathsf {PCon}}}$ *and* $\underline{{\mathsf {IncPC}}}$. Fixing $|\Delta G| = 10\%|G|$, we tested the scalability of parallel $\mathsf {PCon}$ and $\mathsf {IncPC}$ with the number *k* of cores. As shown in Fig. 11k and l on $\mathsf {GSH}$, (1) $\mathsf {PCon}$ scales well with *k*: it is 10.1 times faster when using $k = 20$ cores versus $k=1$ (single core), and it is 4.3 times faster when *k* varies from 4 to 20. (2) $\mathsf {IncPC}$ is on average 1.9 times faster than $\mathsf {PCon}$. (3) $\mathsf {IncPC}$ scales well with *k*; it is 3.7 times faster when *k* varies from 4 to 20, across 4 machines.

The results on other graphs are consistent.

**Summary**. We find the following over 10 real-life graphs. On average, (1) the contraction scheme reduces graphs by 71.9%. The contraction ratio is 0.34, 0.264, 0.213 and 0.365 in social networks, Web graphs, collaboration networks and road networks, respectively. (2) It improves the evaluation of $\mathsf {SubIso}$, $\mathsf {TriC}$, $\mathsf {Dist}$, $\mathsf {CC}$ and $\mathsf {CD}$ by 1.69, 1.44, 1.47, 2.24 and 1.37 times, respectively. Existing algorithms can be adapted to the scheme, with indices or not. (3) On average, contracting the first three types of regular structures improves the efficiency of query evaluation by 1.61, 1.44 and 1.04 times, respectively. (4) Contracting obsolete data improves the efficiency of both conventional queries and temporal queries, by 1.64 and 1.78 times on average, respectively. (5) Its total space cost on $\mathsf {SubIso}$, $\mathsf {TriC}$, $\mathsf {Dist}$, $\mathsf {CC}$ and $\mathsf {CD}$ is only $12.7\%$ of indexing costs of $\mathsf {TurboIso}$, $\mathsf {PLL}$, $\mathsf {HINDEX}$ and $\mathsf {RMC}$. The synopses for the five query classes take only 48.3% of the total space of the contraction scheme. Thus, our contraction scheme scales with the number of applications. (6) Algorithms $\mathsf {GCon}$, $\mathsf {PCon}$, $\mathsf {IncCR}$ and $\mathsf {IncPC}$ scale well with graphs and updates. $\mathsf {GCon}$ takes 344s when *G* has 1.8B edges and nodes, and $\mathsf {PCon}$ takes only 33.1s with 20 cores, across 4 machines. $\mathsf {IncCR}$ is 4.9 times faster than $\mathsf {GCon}$ when $|\Delta G|$ is $5\%|G|$, and is still faster when $|\Delta G|$ is up to $30\%|G|$. (7) $\mathsf {PCon}$ and $\mathsf {IncPC}$ scale well with the number *k* of machines. When $|\Delta G| = 10\%|G|$, $\mathsf {PCon}$ is 4.3 times faster and $\mathsf {IncPC}$ is 3.7 times faster when *k* varies from 4 to 20.

## Related work

This paper extends its conference version [38] as follows. (1) We identify a variety of frequent regular structures in different types of graphs, develop their synopses and contract graphs based on their types (Sect. 2.2). In contrast, [38] adopts an one-size-fit-all solution and contracts only cliques, paths and stars for all types of graphs. (2) In light of new regular structures, all examples and algorithms have been extended (Sects. 2–4). (3) We provide the pseudo code and details of a parallel contraction algorithm (Sect. 2.4). (4) We study two new query classes, namely, (non-local) connected component and (intractable) clique decision, for proof of concept (Sects. 3.4 and 3.5). We also extend the algorithms for the three other cases to cope with newly studied topological components (Sects. 3.1–3.3). (5) We extend the study of incremental contraction by presenting vertex updates and parallel incremental maintenance algorithm (Sect. 4). (6) The experimental study is almost entirely new and evaluates the contraction scheme *w.r.t.*  different regular structures to contract as well as its effectiveness on new big graphs and new query classes of Sects. 3.4 and 3.5 (Sect. 5).

We discuss the other related work as follows.

* Contraction*. As a traditional graph programming technique [43], node contraction merges nodes, and subgraph contraction replaces connected subgraphs with supernodes. It is used in *e.g.,* single source shortest paths [54], connectivity [43] and spanning tree [41].

In contrast, we extend the conventional contraction with synopses to build a compact representation of graphs as a generic optimization scheme, which is a departure from the programming techniques.

* Compression*. Graph compression has been studied for social network analysis [27], community queries [21], subgraph isomorphism [34, 69], graph simulation [37], reachability and shortest distance [50], and GPU-based graph traversal [82]. It often computes query-specific equivalence relations by merging equivalent nodes into a single node or replacing frequent patterns by virtual nodes. Some are query preserving (lossless), *e.g.,* [37, 50, 69], and can answer certain types of queries on compressed graphs without decompression.

Another category of compression aims to minimize the number of bits required to represent a graph. WebGraph [15] exploits the inner redundancies of Web graphs; [8] proposes an encoding scheme based on node indices assigned by the BFS order; [24] approximates the optimal encoding with MinHash; and [52] removes the hub nodes for an scheme to have better locality.

Our contraction scheme differs from graph compression in the following. (a) It optimizes performance of multiple applications with the same contracted graph. In contrast, many compression schemes are query dependent and require different structures for different query classes. While some methods serve generic queries [8, 15, 24], they may incur heavy recovering cost. (b) Contraction is lossless, while some compression schemes are lossy, *e.g.,* [34]. (c) For a number of query classes, their existing algorithms can be readily adapted to contracted graphs, while compression often requires to develop new algorithms *e.g.,* [69] demands a decompose-and-join algorithm for subgraph isomorphism.

* Summarization*. Graph summarization aims to produce an abstraction or summary of a large graph by aggregating nodes or subgraphs (see [67] for a survey), classified as follows. (1) Node aggregation, *e.g.,* $\mathsf {GraSS}$ [60] merges node clusters into supernodes labeled with the number of edges within and between the clusters; it is developed for adjacency, degree and centrality queries. $\mathsf {SNAP}$ [87] generates an approximate summary of a graph structure by aggregating nodes based on attribute similarity. (2) Edge aggregation, *e.g.,* [73] generates a summary by aggregating edges, with a bounded number of edges different from the original graph. (3) Simplification: instead of aggregating nodes and edges, $\mathsf {OntoVis}$ [83] drops low-degree nodes, duplicate paths and unimportant labels. Most summarization methods are lossy, *e.g.,* $\mathsf {GraSS}$ and $\mathsf {SNAP}$ only retain part of attributes, and $\mathsf {OntoVis}$ drops nodes, edges and labels.

Incremental maintenance of summarization has been studied [30, 46, 84]. It depends on update intervals [84]; short-period summarization is space-costly, while long-interval summarization may miss updates. To handle these, [46] aggregates updates into a graph of “frequent” nodes and edges and computes a summary based on all historical updates on entire graph.

Both summarization and contraction schemes aim to provide a generic graph representation to speed up graph analyses. However, contraction differs from summarization in the following. (1) The contraction scheme is *lossless* and returns exact answers for various classes of queries. In contrast, summarization is typically lossy and supports at best certain aggregate or approximate queries only. (2) Many existing algorithms for query answering can be readily adapted to contracted graphs, while new algorithms often have to be developed on top of graph summaries. (3) For a number of query classes studied, contracted graphs can be incrementally maintained with boundedness and locality, while summarization maintenance requires historical updates and often operates on the entire graph [46].

* Indexing*. Indices have been studied for, *e.g.,* subgraph isomorphism [13, 14, 28, 44, 72], reachability [7, 23, 50, 95] and shortest distance [25, 66]. They are query specific, and take space and time to store and maintain.

Our contraction scheme differs from indexing as it supports multiple applications on the same contracted graph, while a separate indexing structure has to be built for each query class. Moreover, it is more efficient to maintain contracted graphs than indices. This said, the contraction scheme can be complemented with indices for further speedup, by building indices on smaller contracted graphs, as demonstrated in Sect. 3.1.

## Conclusion

We have proposed a contraction scheme to make big graphs small, as a generic optimization scheme for multiple applications to run on the same graph at the same time. We have shown that the scheme is generic and lossless. Moreover, it prioritizes up-to-date data by separating it from obsolete data. In addition, existing query evaluation algorithms can be readily adapted to compute exact answers, often without decontracting topological components. Our experimental results have verified that the contraction scheme is effective.

A topic for future work is to build a hierarchy of contracted graphs by iteratively contracting regular structures into supernodes, until the one at the top fits into the memory; the objective is to make large graphs small enough to fit into the memory of a single machine, and make it possible to process large graphs under limited resources. Another topic is to study the capacity of a single multi-core machine for big graph analytics, by leveraging both contraction and multi-core parallelism.
